# The EIF4A3/CASC2/RORA Feedback Loop Regulates the Aggressive Phenotype in Glioblastomas

**DOI:** 10.3389/fonc.2021.699933

**Published:** 2021-08-02

**Authors:** Junshuang Zhao, Yang Jiang, Lian Chen, Yue Ma, Haiying Zhang, Jinpeng Zhou, Hao Li, Zhitao Jing

**Affiliations:** ^1^Department of Neurosurgery, The First Hospital of China Medical University, Shenyang, China; ^2^Department of Neurosurgery, Shanghai Tenth People’s Hospital, Tongji University School of Medicine, Shanghai, China; ^3^Department of Pulmonary and Critical Care Medicine, Shengjing Hospital of China Medical University, Shenyang, China; ^4^International Education College, Liaoning University of Traditional Chinese Medicine, Shenyang, China

**Keywords:** glioblastoma, RORA, CASC2, EIF4A3, TGF-β1/Smad signaling pathway

## Abstract

Glioblastoma (GBM) is a common and refractory subtype of high-grade glioma with a poor prognosis. The epithelial-mesenchymal transition (EMT) is an important cause of enhanced glioblastoma invasiveness and tumor recurrence. Our previous study found that retinoic acid receptor-related orphan receptor A (RORA) is a nuclear receptor and plays an important role in inhibiting proliferation and tumorigenesis of glioma. We further confirmed RORA was downregulated in GBM. Thus, we determined whether RORA was involved in the migration, invasion, and EMT of GBM. Human GBM cell lines, U87 and T98G, and patient-derived glioma stem cells (GSCs), GSC2C and GSC4D, were used for *in vitro* and *in vivo* experiments. The expressions of RORA, CASC2, and EIF4A3 in GBM cells and GSCs were detected by RT-qPCR and western blotting. The biological effects of RORA, CASC2, and EIF4A3 on GBM migration, invasion, and EMT were evaluated using the migration assay, transwell assay, immunofluorescence staining, and xenograft experiments. We found that RORA inhibited the migration, invasion, and EMT of GBM. CASC2 could bind to, maintain the stability, and promote the nuclear translocation of RORA protein. EIF4A3 could downregulate CASC2 expression *via* inducing its cleavage, while RORA transcriptionally inhibited EIF4A3 expression, which formed a feedback loop among EIF4A3/CASC2/RORA. Moreover, gene set enrichment analysis (GSEA) and *in vitro* and *in vivo* experiments showed RORA inhibited the aggressiveness of GBM by negatively regulating the TGF-β1/Smad signaling pathway. Therefore, The EIF4A3/CASC2/RORA feedback loop regulated TGF-β1/Smad signaling pathway might become a promising therapeutic strategy for GBM treatment.

## Introduction

Glioblastoma (GBM) is the most deadly and common tumor in the central nervous system (1, 2). Although there has been more progress made recently in GBM treatment using surgery, radiotherapy, and chemotherapy, the therapeutic effects are still not completely satisfactory (3). It has been reported that the 5-year survival is limited to 5% (1, 4). Molecular targeting therapy may be one of the most promising therapies for GBM (5). Therefore, identifying genes that play an important role in GBM may provide new options for treating this disorder.

Retinoic acid receptor-related orphan receptor A (RORA) is a type of nuclear receptor, which is also a transcription factor and binds to the promoter of target genes to regulate the expression and structural changes of target genes (6). Previous studies have shown downregulations of RORA expression in various tumor tissues, such as breast, lung, prostate, and colorectal cancer (7). It has been reported that the epithelial-mesenchymal transition (EMT) is an important cause of enhanced GBM invasiveness and tumor recurrence (8). Our previous study showed that RORA played an anti-tumor role and inhibited the tumorigenesis and proliferation in glioma (9). In the present study, the possible role and effects of RORA in the migration, invasion, and EMT of GBM were identified.

TGF-β/Smad signaling pathway is one of the most important signaling pathway regulating EMT in GBM. It is activated in high-grade gliomas and associated with patients’ poor prognosis (10, 11). Besides, TGF-β signaling pathway was also reported as a potential signature for the mesenchymal subtype of GBM (12). Our study found that the TGF-β signaling pathway was enriched with RORA expression according to gene set enrichment analysis (GSEA), suggesting that RORA might regulate the EMT of GBM *via* TGF-β/Smad signaling.

Long non-coding RNAs (lncRNAs) are defined as a series of RNA polymerase II transcripts with a length of more than 200 nucleotides, which are involved in various genetic phenomena, such as transcriptional, post-transcriptional, and epigenetic regulations (13–15). LncRNAs have been regarded as a modulator in the induction and progression of GBM (16). Cancer susceptibility candidate 2 (CASC2) is a lncRNA-encoding gene of humans and is located on chromosome 10q26 (17). It has been reported that the low expression of lncRNA CASC2 in glioma serves as a tumor suppressor (18). Bioinformatics analysis predicted that CASC2 might bind to RORA proteins. However, there are limited reports about whether CASC2 regulated the expression of RORA in GBM.

RNA-binding proteins (RBPs) are a series of specific proteins, which could bind to targeted RNA and regulate its processing, localization, transport, and stability (19). Eukaryotic initiation factor 4A3 (EIF4A3) is a member of the DEAD-box RNA helicase family and regarded as one of the special components of the exon junction complex (EJC). EIF4A3 is involved in various RNA metabolic processes such as nonsense-mediated RNA decay (NMD) and RNA splicing (20, 21). It has been reported that EIF4A3 promoted the malignant biological processes of glioblastoma cells *via* stabilizing LINC00680 and TTN-AS1 (22). This study predicted that EIF4A3 is the most possible candidate RBPs for CASC2 according to the Starbase database. However, the potential mechanisms between EIF4A3 and CASC2 on the biological processes of GBM have not been illustrated.

In the present study, we first found that RORA expressed lower in GBM specimens, which meant the poorer prognosis of GBM patients. RORA overexpression inhibited the migration, invasion, and EMT of GBM *via* inactivating TGF-β1/Smad signaling pathway *in vitro* experiments. Furtherly, CASC2 could bind to, regulate the stability and promote the nuclear translocation of RORA protein. Besides, EIF4A3 could downregulate the expression of CASC2 *via* inducing its cleavage. Moreover, RORA also transcriptionally inhibited the expression of EIF4A3, which formed a feedback loop among EIF4A3/CASC2/RORA. Thus, we aimed to confirm whether the EIF4A3/CASC2/RORA feedback loop played an important role in GBM progression.

## Materials and Methods

### Cell Culture and Treatment

Normal human astrocytes were purchased from ScienCell Research Laboratories (San Diego, CA, USA) and cultured in astrocyte medium (ScienCell Research Laboratories, Carlsbad, CA, USA). The human GBM cell lines LN229 and T98G were purchased from the American Type Culture Collection (Manassas, VA, USA). The human GBM cell lines, U178 and H4, were purchased from iCell Bioscience (Shanghai, China). The human GBM cell lines, U87, U118, and U251, were purchased from the Chinese Academy of Sciences cell bank (Shanghai, China). All GBM cell lines were cultured in Dulbecco’s Modified Eagle’s Medium (DMEM; HyClone, Logan, UT, USA), supplemented with 10% fetal bovine serum (FBS; Gibco, Carlsbad, CA, USA) and 1% penicillin/streptomycin (Gibco) at 37°C with 5% CO_2_. Six patient-derived primary glioma stem cells (GSCs) with World Health Organization (WHO) grade II, III, and IV (WHO grade II: GSC2C and GSC2D; III: GSC3C and GSC3D; and IV: GSC4C and GSC4D) were cultured as previously described (23). The detailed clinicopathological information is presented in **Supplementary Table 1**. The stemness of GSCs was detected by immunofluorescence staining of CD133 and nestin (Abcam, Cambridge, UK) and the multi-lineage differentiation capacity of GSCs was detected by immunofluorescence staining of GFAP and β III tubulin (Abcam). Human recombinant TGF-β1 (Abcam) was used as a standard at a concentration of 5 ng/mL.

### Patients and Samples

Eighty-seven clinical samples from glioma patients were collected from January 2007 to January 2012 at the First Affiliated Hospital of China Medical University. There were 45 lower-grade gliomas and 42 glioblastomas. During the same period, 18 acute brain injury samples from patients were collected as the control group. Clinical information for these samples is provided in **Supplementary Table 2**. This study received the approval of the Ethics Committee of the First Affiliated Hospital of China Medical University, and each patient signed an informed consent form.

### Lentiviral Vector Construction and Transfection

The lentivirus transfection was performed as previously described (23). The lentivirus-based vectors for RORA, CASC2, and EIF4A3 overexpression, the RNAi mediated knockdown of RORA, CASC2, EIF4A3, and its negative control were all acquired from Gene-Chem (Shanghai, China). The sequences of all siRNAs are listed in **Supplementary Table 3**. The transfection efficacy was detected by RT-qPCR and western blotting.

### RT-qPCR

RT-qPCR was performed as previously described (23). The Mini-BEST Universal RNA Extraction kit (TaKaRa, Kyoto, Japan) was used to extract the total RNA of glioma cells. Then, the Prime-Script RT Master Mix (TaKaRa) was used for first-strand cDNA synthesis. Finally, the RT-qPCR assays were detected using the SYBR Green Master Mix (TaKaRa) *via* a PCR LightCycler480 (Roche Diagnostics, Basel, Switzerland). Primers used in this study are listed in **Supplementary Table 4**.

### Western Blotting

Western blotting was performed as previously described (23). A total cell protein extraction kit (KeyGen Biotechnology, Nanjing, China) was used to isolate the total proteins of GBM cells or tissues, followed by electrophoresis and transferring to a nitrocellulose membrane, then blocked for 2 h at room temperature with 2% bovine serum albumin (KeyGen Biotechnology). The membranes were incubated overnight at 4°C with the following primary antibodies against: RORA (1:1,000; Abcam), EIF4A3 (1:1,000; Abcam), TGF-β1 (1:1,000; Abcam), p-SMAD2 (1:1,000; Cell Signaling Technology, Danvers, MA, USA), SMAD2 (1:1,000; Cell Signaling Technology), p-SMAD3 (1:1,000; Cell Signaling Technology), SMAD3 (1:1,000; Cell Signaling Technology), Snail (1:1,000; Cell Signaling Technology), Slug (1:1,000; Cell Signaling Technology), E-cadherin (1:2,000; Abcam), vimentin (1:2,000; Abcam), N-cadherin (1:2,000; Abcam), p-AKT (1:1,000; ProteinTech, Chicago, IL, USA), AKT (1:1,000; ProteinTech), p-ERK (1:2,000; ProteinTech), ERK (1:2,000; ProteinTech), p-JNK (1:3,000; ProteinTech), JNK (1:3,000; ProteinTech), p-p38 (1:1,000; ProteinTech), p38 (1:1,000; ProteinTech), and β-actin (1:2,000; ProteinTech). Following a 2 h treatment with secondary antibodies (ProteinTech, Rosemont, IL, USA), all bands were detected using a chemiluminescence ECL kit (Beyotime Biotechnology, Beijing, China) and quantified by ImageJ software (National Institutes of Health, Bethesda, MD, USA). The relative expression was calculated using β-actin as the internal control.

### Immunohistochemistry (IHC)

IHC was performed as previously described (23). First, the tumor tissues were embedded in paraffin, sliced into 4 mm sections, and labeled with the following primary antibodies against RORA (1:100; Abcam), TGF-β1 (1:100; Abcam), E-cadherin (1:100; Abcam), and vimentin (1:100; Abcam). Then, the slices were stained with an immunohistochemical labeling kit (MaxVision Biotechnology, Fuzhou, China) and imaged using light microscopy (Olympus, Tokyo, Japan). Finally, the staining intensities and the expression levels were evaluated according to the German immunohistochemical score (10).

### Immunofluorescence

Immunofluorescence staining was performed as previously described (23). First, the GBM cells were fixed with 4% paraformaldehyde (Solarbio, Beijing, China) for 10 min, permeabilized with 0.5% Triton X-100 (Solarbio) for 20 min, blocked with 5% bovine serum albumin (Solarbio) for 1 h, and probed with primary antibodies to CD133, nestin, GFAP, βIII-tubulin, E-cadherin, and vimentin (1:100; Abcam) at 4°C overnight. Then, all GBM cell samples were treated with fluorescein isothiocyanate or rhodamine-conjugated secondary antibodies. Subsequently, the cells were counterstained with 4ʹ,6-diamidino-2-phenylindole (Sigma-Aldrich, Shanghai, China). Finally, the staining was visualized using a laser scanning confocal microscope (Olympus).

### Cell Migration and Transwell Assays

For the migration assay, the cells were resuspended in serum-free medium (HyClone) at a density of 2 × 10^5^ cells/mL, and then 100 μL of cell suspension was seeded into the upper chamber (Costar, Corning, NY, USA), and 600 μL DMEM/high glucose medium (HyClone) with 10% FBS was added to the lower chamber. After incubation at 37°C for 24 h, the cells were fixed with 4% paraformaldehyde (Solarbio) for 10 min and stained with 1% Crystal Violet solution (Solarbio) for 20 min in room temperature. Finally, the cell numbers were counted by calculating the average of five random fields using an inverted microscope (Olympus). For the Transwell assay, the 8 μm pore size polycarbonate membrane was covered with 80 μL of 50 ng/μL Matrigel solution (BD, Franklin Lakes, NJ, USA). The other steps were the same as the migration assay.

### RNA Immunoprecipitation (RIP) Assay

According to the manufacturer’s instructions, the RIP assay was performed *via* the Imprint RNA Immunoprecipitation Kit (Sigma, USA). All GSCs lysates under different conditions were incubated with RIP buffer, including magnetic beads conjugated with the negative control IgG, anti-RORA, or anti-EIF4A3 antibodies (Millipore, UK). The immunoprecipitated RNAs were acquired after incubated with Proteinase K buffer (Omega, Shanghai, China). Finally, the precipitants were detected using RT-qPCR.

### RNA Pull-Down Assay

The RNA pull-down assays were performed as previously described (24). The Pierce Magnetic RNA Protein pull-down Kit (Thermo Fisher Scientific) was used according to the manufacturer’s instructions. Briefly, the biotinylated RNA probes were used to label the purified RNA, and then the biotinylated RNA, the negative control (antisense RNA), and the positive control (input) were mixed and co-incubated with proteins of GSCs. To prepare a probe-magnetic bead complex, the RNA-protein complex was added with magnetic beads. After being washed and boiled, the complexes were detected by western blotting, and β-actin was used for the control.

### Nascent RNA Capture

All nascent RNAs were detected using the Click-iT nascent RNA capture kit (Thermo Fisher Scientific, USA) according to the manufacturer’s instructions. Briefly, all nascent RNAs were treated with 5-ethynyl uridine (EU), then streptavidin magnetic beads were used to capture the EU-nascent RNAs and finally detected *via* RT-qPCR.

### RNA Stability Evaluation

Actinomycin D (ActD; NobleRyder, China) was added into the cell culture medium to restrain the *de novo* synthesis of RNA. Then, at different time points, total RNA was isolated and detected by RT-qPCR. Finally, at a certain time point, the half-life of RNA was confirmed by its level decreasing to 50%.

### Luciferase Reporter Assay

Luciferase reporter assays were performed as described previously (25). Firstly, the EIF4A3 and TGF-β1 reporter plasmids were constructed by Gene-Chem. The predicted 3’-UTR sequences of EIF4A3 and TGF-β1 and their corresponding mutant sequence were cloned into pGL3 Dual-Luciferase Vector to construct the luciferase reporter vectors (EIF4A3-Wt or EIF4A3-Mut, TGF-β1-Wt or TGF-β1-Mut). Then, the GSCs were seeded into 96-well plates at a density of 5 × 10^3^ cells/well, transfected with EIF4A3-Wt or EIF4A3-Mut, TGF-β1-Wt or TGF-β1-Mut reporter plasmids, and incubated for 48 h. Finally, the luciferase activities were detected using a Dual-Luciferase Reporter Assay System (Promega, Madison, WI, USA).

### Enzyme-Linked Immunosorbent Assay (ELISA)

ELISAs were performed as previously described (23). The human TGF-β1 Quantikine ELISA Kit (R&D Systems, Minneapolis, MN, USA) was used to detect the concentrations of TGF-β1 in the media supernatants of the GBM cells and GSCs. All ELISA readings were normalized to the protein concentration in the control groups.

### Xenograft Experiments

Xenograft experiments were conducted according to the Animal Care Committee of China Medical University. Six-week-old male BALB/c nude mice (Beijing Vital River Laboratory Animal Technology, Beijing, China) were divided into six groups: control, RORA-OE, CASC2-KD1, CASC2-KD1+RORA-OE, EIF4A3-OE, and EIF4A3-OE+RORA-OE. Each group with five mice was bred in the Laboratory Animal Center of China Medical University under specific pathogen-free conditions. The GSC4D treated with different conditions was orthotopically injected into the mouse brain at 2 mm lateral and 2 mm anterior to the bregma using a stereotaxic apparatus (5 × 10^4^ cells each mouse). Each group was observed daily for distress or death signs. The mice were sacrificed, the tumors isolated, and the tumor volume was calculated according to the formula: V = (D × d^2^)/2, (D was the longest diameter and d was the shortest diameter). The overall survival times of mice were detected through Kaplan-Meier survival analysis.

### Bioinformatics Analysis

The data on RORA mRNA expressions of glioma patients were obtained from the Chinese Glioma Genome Atlas (CGGA, http://www.cgga.org.cn) using the RNA-seq platform and The Cancer Genome Atlas (TCGA, http://cancergenome.nih.gov) in HG-U133A platforms. GSEA (http://www.broadinstitute.org/gsea/index.jsp) was used to detect the enrichment of signaling pathways between the high and low RORA expression groups. Starbase (http://starbase.sysu.edu.cn) was used to predict the relative lncRNAs by examining the lncRNAs and RORA 3’-UTR using bioinformatics algorithms.

### Statistical Analysis

All experiments were repeated at least three times, and the results are expressed as the mean ± SD using SPSS statistical software for Windows, version 22.0 (SPSS, Chicago, IL, USA). Comparisons of two independent groups were determined using the chi-square test and two-tailed Student’s t-test. The statistical significance among three or more groups was evaluated by one-way analysis of variance. Pearson’s correlation analysis was used to detect the correlation between the two groups. Kaplan-Meier analysis and the log-rank test were conducted to analyze the survival rates of each group. Two-tailed P values < 0.05 were considered significant.

## Results

### RORA Overexpression Predicts Better Prognoses in GBM Patients

**Figure 1:**
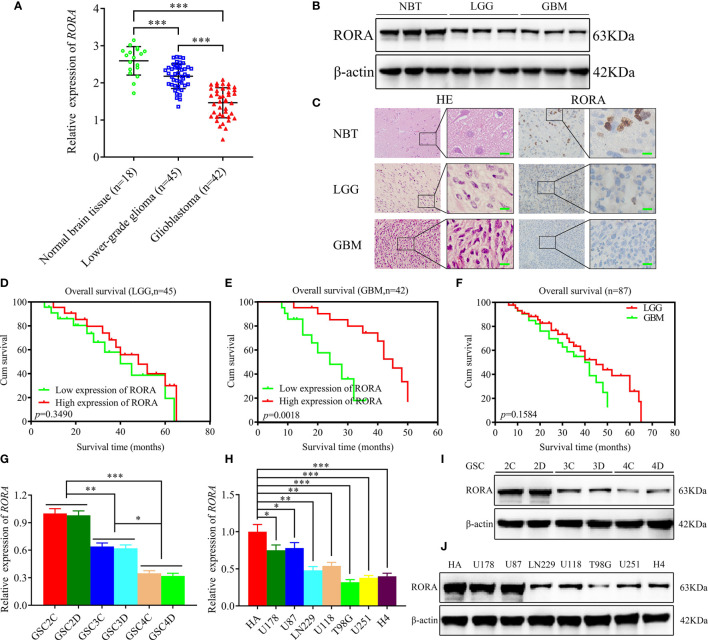
Retinoic acid receptor-related orphan receptor A (RORA) has low expression in glioblastomas and correlates with poorer patient survival, and the expression of RORA in patient-derived GSCs and GBM cell lines. **(A–C)** RT-qPCR **(A)**, western blotting **(B)**, and immunohistochemistry analyses **(C)** showing RORA expressions in different clinical glioma specimens, when compared with normal brain tissues (NBTs). β-actin was used as a loading control. (lower-grade glioma, LGG, n = 45; glioblastoma, GBM, n = 42; NBTs, n = 18; *P* < 0.001; one-way analysis of variance). Scale bar = 50 µm. **(D–F)** Kaplan-Meier analysis of 87 cases of glioma patients with high RORA expressions *versus* low RORA expressions. **(G–J)** RT-qPCR **(G, H)** and western blotting **(I, J)** showing RORA expression in six patient-derived GSCs and in GBM cell lines. All data are expressed as the mean ± SD (three independent experiments). ^*^P < 0.05; ^**^P < 0.01; ^***^P < 0.001.

We first detected the expressions of RORA in 87 clinical glioma specimens and 18 normal brain tissues using RT-qPCR, western blotting, and immunohistochemical staining (**Figures 1A–C**). These results showed that RORA expressed lowest in GBM, followed with lower-grade glioma, and highest expression in normal brain tissues. Besides, we further conducted a Kaplan-Meier survival analysis to evaluate whether the expressions of RORA affected the prognoses of glioma patients (**Figures 1D–F**). The results showed that RORA significantly impacted survival in GBM patients. However, RORA had no significant effect on the prognoses of lower-grade glioma patients.

### The Expression of RORA in Patient-Derived Glioma Stem Cells (GSCs) and GBM Cell Lines

We successfully cultured six glioma stem cell lines derived from GBM patients. Afterward, the six GSCs populations were designated GSC2C and GSC2D (WHO grade II), GSC3C and GSC3D (WHO grade III), and GSC4C and GSC4D (WHO grade IV). All GSCs were confirmed using immunofluorescence staining of stem cell markers, CD133 and nestin (**Figure S1A**), and differentiation markers, GFAP and β-III tubulin (**Figure S1B**). Both RT-qPCR and western blotting were performed to detect the expressions of RORA in GSCs and common GBM cell lines and normal human astrocytes. The results showed that RORA expression in GSC2C was the highest and in GSC4D were the lowest. We also found that the expressions of RORA in common GBM cell lines were lower than those in normal human astrocytes (**Figures 1G–J**).

### RORA Regulates the Migration, Invasion, and EMT of GBM

**Figure 2:**
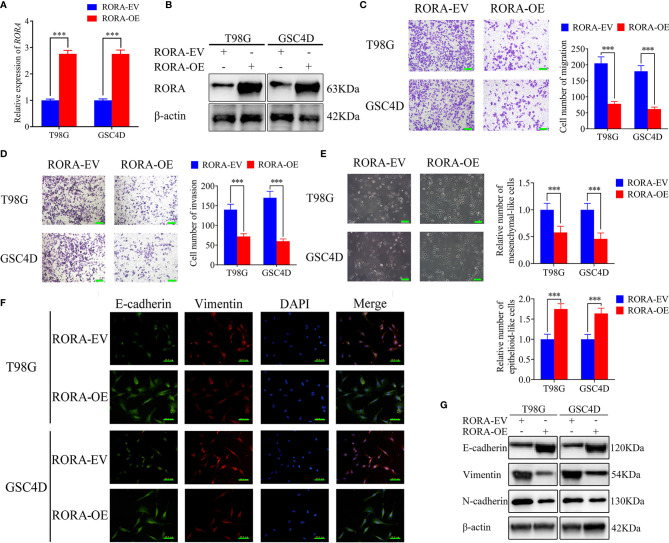
RORA inhibits the migration and invasion of GBM cells *in vitro* and blocks the epithelial-mesenchymal transition (EMT) in GBM. **(A, B)** qPCR **(A)** and western blotting **(B)** showing the validation of RORA overexpression. **(C, D)** Representative migration assay and transwell assay showing the migration rates and invasion rates of T98G cells and GSC4D with RORA overexpression, and the negative control. Scale bar = 100 μm. **(E)** Representative microphotographs showing the morphological changes in RORA-overexpressed T98G cells and GSC4D. Scale bar = 50 μm. **(F, G)** Representative immunofluorescence staining **(F)** and western blotting **(G)** showing the changes in E-cadherin, vimentin, and N-cadherin in T98G cells and GSC4D after RORA overexpression. Scale bar = 50 μm. EV, empty vector; OE, overexpression. All data are expressed as the mean ± SD (three independent experiments). ^***^P < 0.001.

Since there was significantly low RORA expression in GBMs, we predicted that RORA might be associated with the aggressiveness of GBMs. U87 cells and GSC2C with the highest RORA expression were used for RORA knockdown, while T98G cells and GSC4D were chosen for RORA overexpression (**Figures 1G–J**). Both RT-qPCR and western blotting were performed to validate RORA overexpression (**Figures 2A, B**). We further detected the migration, invasion, and EMT in RORA-overexpressed T98G cells and GSC4D by migration assays, transwell assays, phase contrast microscope, immunofluorescence staining, and western blotting. All results showed that the migration rates, invasion rates, the relative number of mesenchymal-like cells, expression levels of vimentin and N-cadherin significantly decreased, and the relative number of epithelioid-like cells and E-cadherin expression increased (**Figures 2C–G**). However, we obtained the opposite results in RORA-silenced U87 cells and GSC2C (**Figures S2A–G**).

### RORA Negatively Affects the TGF-β1/Smad Signaling Pathway

**Figure 3:**
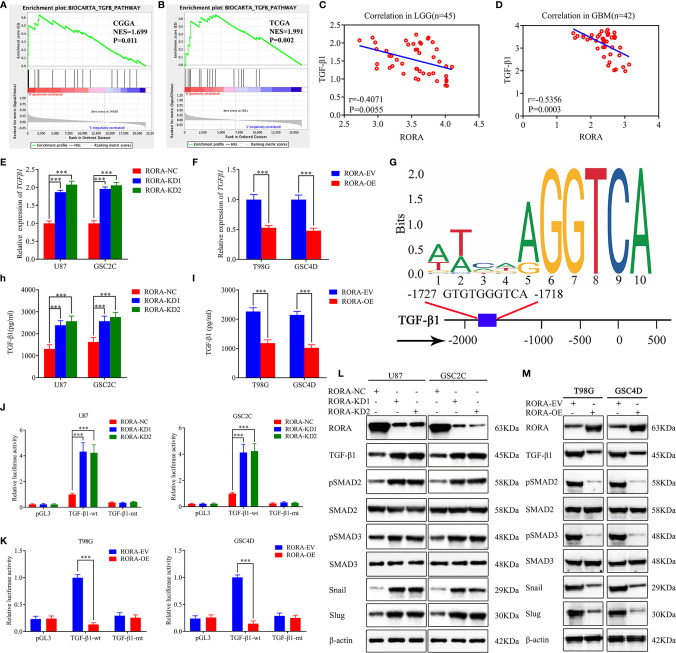
RORA regulates the migration, invasion, and EMT of GBM by negatively affecting the TGF-β1/Smad signaling pathway. **(A, B)** GSEA showing high expression of RORA correlates with the TGF-β signaling pathway in both the Chinese Glioma Genome Atlas and The Cancer Genome Atlas databases. **(C, D)** The relative expression correlation between RORA and TGF-β1 in 87 cases of glioma patients was detected by RT-qPCR. **(E, F)** and **(H, I)** The expression and secretion of TGF-β1 after RORA knockdown **(E, H)** or overexpression **(F, I)** was detected by RT-qPCR **(E, F)** and ELISA **(H, I)**. **(G)** Sequence motif representing the consensus RORA binding motif (JASPAR database), and Schematic representation of the human TGF-β1 promoter region. **(J, K)** RORA knockdown **(J)** or overexpression **(K)** altered the luciferase promoter activities of TGF-β1. **(L, M)** Western blotting showing that the downstream targets of the TGF-β1/Smad signaling pathway were regulated after RORA knockdown **(L)** or overexpression **(M)**. NC, negative control; KD, knockdown; EV, empty vector; OE, overexpression. All data are expressed as the mean ± SD (three independent experiments). ^***^P < 0.001.

To identify the specific signaling pathway involved in RORA-regulated migration, invasion, and EMT of GBM, we conducted a GSEA based on the CGGA and TCGA databases and found that RORA expression was associated with the TGF-β signaling pathway (**Figures 3A, B**). The correlations between RORA and TGF-β1 expressions in 87 cases of clinical glioma specimens were then detected by RT-qPCR, which showed significant negative correlations in lower-grade gliomas and GBMs (**Figures 3C, D**). Then, we detected the expressions of TGF-β1 after RORA knockdown or overexpression by RT-qPCR, western blotting, and ELISA assays. All results showed that the expressions of TGF-β1 were upregulated in RORA-silenced U87 cells and GSC2C, while the opposite results were found in RORA-overexpressed T98G cells and GSC4D (**Figures 3E, F, H, I, L, M**). We further detected the downstream proteins of the TGF-β1/Smad signaling pathway by western blotting and found that the expression of p-SMAD2, p-SMAD3, Snail, and Slug were significantly upregulated in RORA-silenced U87 cells and GSC2C (**Figure 3L**). We obtained the opposite results in RORA-overexpressed T98G cells and GSC4D (**Figure 3M**). Besides, we also performed western blotting to detect the non-canonical signaling of TGF-β1, including MAPK pathways, JNK, p38 and PI3K cascade. The results showed there was no change in the expression levels of p-ERK, p-JNK, p-p38 and p-AKT after RORA overexpression (**Figure S3**). Because RORA is a transcription factor, we investigated whether RORA transcriptionally regulated TGF-β1 expression (**Figure 3G**). We performed luciferase reporter assays and found that the relative luciferase activities of TGF-β1 significantly increased in RORA-silenced U87 cells and GSC2C; however, the opposite results were obtained in RORA-overexpressed T98G cells and GSC4D (**Figures 3J, K**). Taken together, RORA negatively affected the TGF-β1/Smad signaling pathway.

### Recombinant TGF-β1 Treatment Abrogates the Inhibiting Effects of RORA on the Migration, Invasion, and EMT of GBM

To confirm whether RORA inhibited the migration, invasion, and EMT of GBM *via* negatively affecting the TGF-β1/Smad signaling pathway, the RORA-overexpressed GSC4D was treated with human recombinant TGF-β1. Both the migration assays and the transwell assays found that RORA overexpression-induced migration and invasion inhibition was reversed after TGF-β1 treatment (**Figures S4A, B**). We then used phase-contrast microscopy to observe the cell morphology and found that RORA-overexpressed GSC4D showed more mesenchymal-like morphology after TGF-β1 treatment. However, the epithelioid-like morphology decreased after TGF-β1 treatment (**Figure S4C**). Subsequently, we detected E-cadherin, vimentin, and N-cadherin by immunofluorescence staining and western blotting (**Figures S4D, E**). The results showed that E-cadherin expression decreased and vimentin and N-cadherin expression increased in RORA-overexpressed GSC4D after TGF-β1 treatment. Besides, the expression of p-SMAD2, p-SMAD3, Snail, and Slug were also upregulated in RORA-overexpressed GSC4D after TGF-β1 treatment. In summary, RORA inhibited the migration, invasion, and EMT of GBM *via* inhibiting the TGF-β1/Smad signaling pathway. Moreover, we also explored whether RORA inhibited the proliferation of GBM *via* TGF-β1/Smad signaling pathway. Both MTS and EDU assays confirmed that RORA can inhibit the proliferation of GBM and these inhibiting effects were reversed after TGF-β1 treatment (**Figures S5A, B**).

### CASC2 Binds to and Maintains the Stability of the RORA Protein

**Figure 4:**
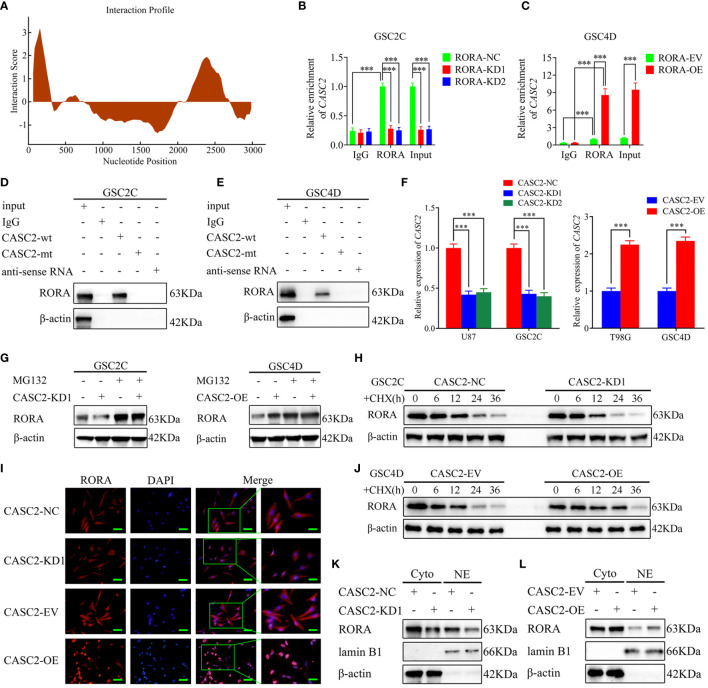
LncRNA CASC2 binds to and maintains the stability of RORA protein and promotes the nuclear translocation of RORA. **(A)** CatRAPID predicts that CASC2 can bind to RORA proteins. **(B, C)** The RNA immunoprecipitation (RIP) assay was performed in GSC2C or GSC4D after RORA knockdown or overexpression, and negative control was transfected, followed by q-PCR to detect the enrichment of CASC2 and RORA. **(D, E)** The RNA pull-down assays showed the RORA protein immunoprecipitation with CASC2 as detected by western blotting. **(F)** qPCR showing the validation of CASC2 knockdown or overexpression. **(G)** CASC2-silenced GSC2C, and CASC2-overexpressed GSC4D and their control were treated with or without MG132 (50 µM) for 6 h, then, cell lysates were detected by western blotting with indicated antibodies, β-actin was used as the control. **(H, J)** CASC2-silenced GSC2C, and CASC2-overexpressed GSC4D were treated with cycloheximide (CHX, 100 ng/ml) to indicate periods of time, and cell lysates were detected by western blotting to indicate the half-life of RORA protein. **(I)** Representative images of subcellular localization of RORA in CASC2-silenced GSC2C and CASC2-overexpressed GSC4D were shown by immunofluorescence. Scale bars=50 µm. **(K, L)** Nuclear and cytosolic lysates were extracted from CASC2-silenced GSC2C and CASC2-overexpressed GSC4D, followed by western blotting with indicated antibodies. EV, empty vector; OE, overexpression; NC, negative control; KD, knockdown. All data are expressed as the mean ± SD (three independent experiments). ^***^P < 0.001.

Increasing evidence has revealed that lncRNAs in the cytoplasm may conduct as decoys for miRNAs or proteins (26). The expression level of lncRNA CASC2 is downregulated in glioma, which is similar to our previous research that CASC2 serves as a tumor suppressor. Moreover, we have certified that RORA mRNA and protein were also downregulated in glioblastoma. Therefore, we further studied the relationship between CASC2 and RORA in GBM. It was predicted that CASC2 could bind to RORA proteins according to catRAPID (**Figure 4A**). Then RIP assays were performed and indicated an obvious enrichment of CASC2 co-precipitated within RORA immunocomplex. The relative enrichment of CASC2 in the anti-RORA group was obviously decreased after RORA knockdown but increased after RORA overexpression. However, the relative enrichment of CASC2 in the IgG-treated group showed no significant changes (**Figures 4B, C**). Moreover, RNA pull-down assays also showed that CASC2 could bind to RORA protein in both GSC2C and GSC4D (**Figures 4D, E**). Besides, we performed RT-qPCR to validate the efficiency of CASC2 knockdown or overexpression (**Figure 4F**). Our study also certified that CASC2 could upregulate the expression of RORA protein according to the western blotting (**Figures S6A, B**). Surprisingly, we found that RORA mRNA expression had no significant change after CASC2 overexpression or knockdown *via* RT-qPCR (**Figures S6C, D**).

Next, we tried to explore the possible molecular function between CASC2 and RORA protein. Since the protein levels in tumor tissues could be regulated by transcription, mRNA translation, protein stability, or proteasome-mediated degradation (27), we speculated that CASC2 might upregulate RORA protein expression by maintaining the protein stability. Notably, the reduced protein level of RORA by CASC2 knockdown was apparently recovered after MG-132 treatment in GSC2C, while the opposite results were obtained in CASC2-overexpressed GSC4D (**Figure 4G**). Moreover, cycloheximide (CHX) chase assay demonstrated that RORA in CASC2-silenced GSC2C showed shorter half-life, while the half-life of RORA was much longer in CASC2-overexpressed GSC4D than that in controls (**Figures 4H, J**), suggesting that the regulation of RORA protein by CASC2 might through inhibiting proteasome degradation. Taken together, these results suggested that CASC2 could bind to RORA proteins and actively regulate the expression of RORA proteins.

### CASC2 Promotes the Nuclear Translocation of RORA

Since lncRNA can regulate the distribution of targeted genes in the nucleus and cytoplasm, the distribution of RORA in GSCs was observed *via* immunofluorescence staining (**Figure 4I**). The results showed that RORA levels presented in the nucleus and cytoplasm were obviously decreased in CASC2-silenced GSC2C, while an increased RORA level was presented after CASC2 overexpression in GSC4D. Then western blotting analysis of nuclear and cytosolic fractions was performed, and similar results were also obtained (**Figures 4K, L**). CASC2 overexpression can upregulate RORA expression in nuclear and cytosolic, while the opposite results were obtained after CASC2 knockdown. Together, these results suggested that CASC2 could promote the nuclear translocation of RORA.

### CASC2 Inhibits the Migration, Invasion, and EMT of GBM, and the Inhibiting Effects Are Reversed by RORA Knockdown

**Figure 5:**
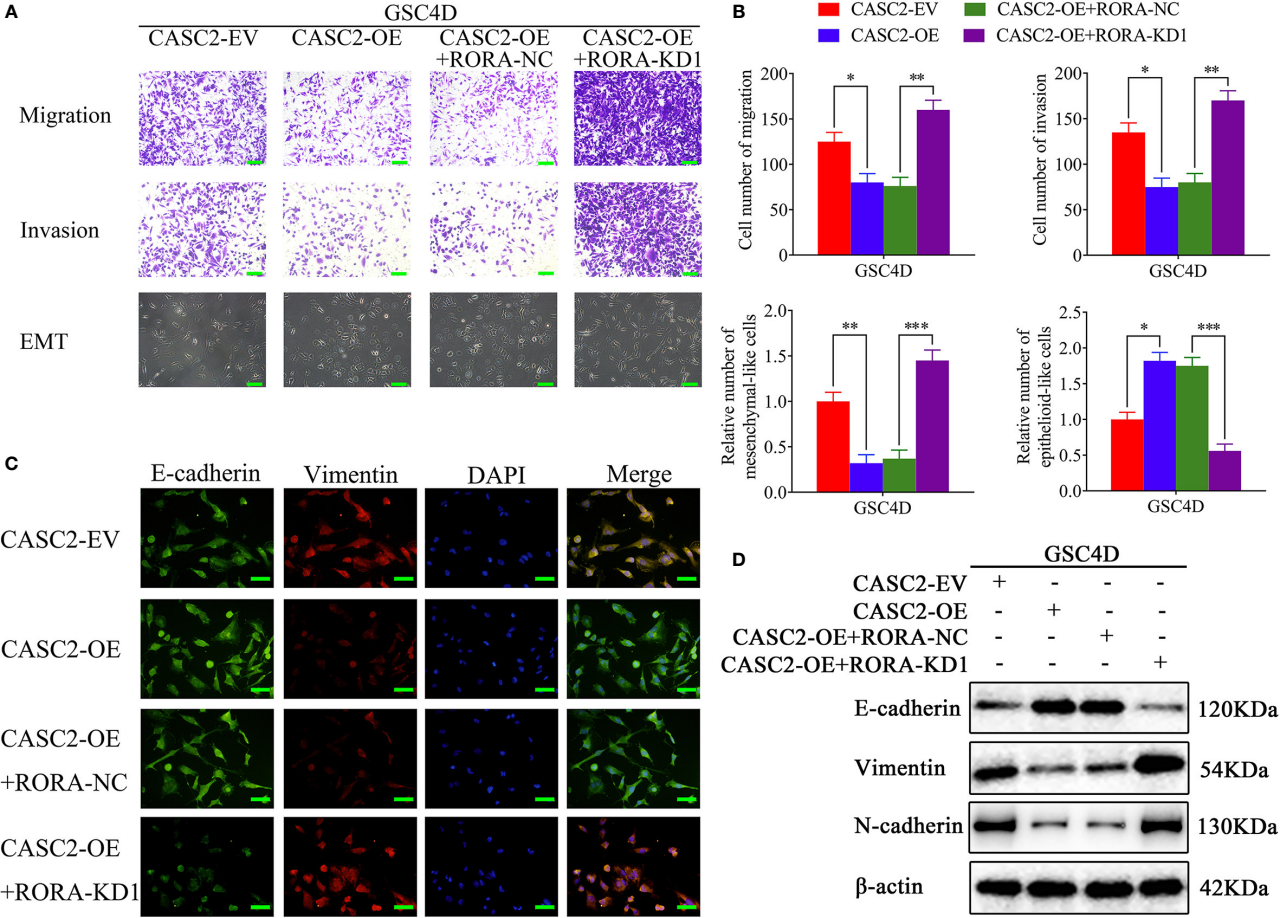
CASC2 inhibits the migration, invasion, and EMT of GBM, and the inhibitory effects are reversed by RORA knockdown. **(A, B)** Representative migration assays and Transwell assays showing the migration rates and invasion rates, and representative microphotographs showing the morphological changes in CASC2-overexpressed GSC4D, reversed by RORA knockdown, and the negative control. The migration assay and Transwell assay: scale bar = 100 μm, microphotographs: scale bar = 50 μm. **(C**, **D)** Representative immunofluorescence staining **(C)** and western blotting **(D)** showing the changes in E-cadherin, vimentin, and N-cadherin in GSC4D with CASC2 overexpression, reversed by RORA knockdown. Scale bar = 50 μm. EV, empty vector; OE, overexpression; NC, negative control; KD, knockdown. All data are expressed as the mean ± SD (three independent experiments). ^*^P < 0.05; ^**^P < 0.01; ^***^P < 0.001.

Previous studies have confirmed that CASC2 functions as a suppressor for glioma and could inhibit glioma cell proliferation (28, 29). To further confirm that CASC2 inhibited the migration, invasion, and EMT of GBM, we first detected the migration, invasion, and EMT in the CASC2-overexpressed GSC4D. All results showed that the migration rates, invasion rates, the relative number of mesenchymal-like cells, the expression levels of vimentin and N-cadherin significantly decreased, and the relative number of epithelioid-like cells and E-cadherin expression were increased in CASC2-overexpressed GSC4D, while the inhibitory effects of CASC2 overexpression were reversed after RORA knockdown *via* rescue experiments (**Figures 5A–D**). In summary, CASC2 inhibited the migration, invasion, and EMT of GBM, and the inhibiting effects were restrained following RORA knockdown.

### EIF4A3 Downregulates the Expression of CASC2 *via* Inducing Its Cleavage

**Figure 6:**
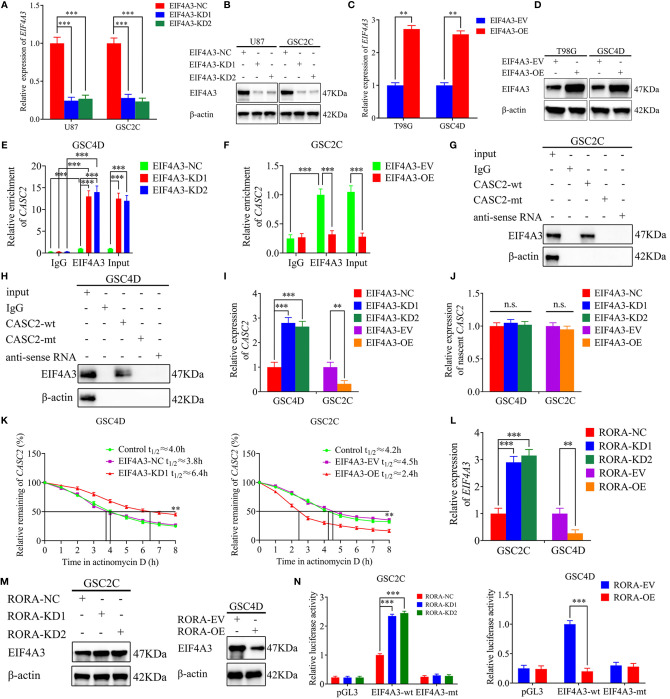
EIF4A3 downregulates the expression of CASC2 *via* inducing its cleavage, while RORA transcriptionally regulates the expression of EIF4A3. **(A–D)** qPCR **(A, C)** and western blotting **(B, D)** showing the validation of EIF4A3 knockdown or overexpression. **(E, F)** The RNA immunoprecipitation (RIP) assay was performed in GSC4D or GSC2C after EIF4A3 knockdown or overexpression, and negative control was transfected, followed by q-PCR to detect the enrichment of CASC2 and EIF4A3. **(G, H)** The RNA pull-down assays showed the EIF4A3 protein immunoprecipitation with CASC2 as detected by western blotting. **(I)** RT-qPCR showing the expression of CASC2 after EIF4A3 knockdown or overexpression. **(J)** The nascent CASC2 was detected by RT-qPCR. **(K)** Graphs showing CASC2 levels at different times treated by actinomycin D in GSC4D and GSC2C. **(L)** RT-qPCR showing the expression of EIF4A3 after RORA knockdown or overexpression. **(M)** Western blotting showing the expression of EIF4A3 protein in RORA-silenced GSC2C and RORA-overexpressed GSC4D. **(N)** The luciferase reporter assays showed that RORA knockdown or overexpression affected the luciferase activities of EIF4A3 in GSCs. EV, empty vector; OE, overexpression; NC, negative control; KD, knockdown. All data are expressed as the mean ± SD (three independent experiments). ^**^P < 0.01; ^***^P < 0.001; n.s., not significant.

RBPs are a special protein that can bind to lncRNA and regulate its processing, transport, localization, and stability (30). We found that EIF4A3 was the most probable RBP to interact with CASC2 according to the Starbase database. Firstly, we performed RT-qPCR and western blotting to validate EIF4A3 silencing or overexpression (**Figures 6A–D**). Then RIP assay showed that the enrichment of CASC2 was significantly increased in the anti-EIF4A3 group compared with the negative control anti-IgG group. The relative enrichment of CASC2 in the anti-EIF4A3 group was obviously increased after EIF4A3 knockdown but decreased after EIF4A3 overexpression. However, the relative enrichment of CASC2 in the IgG-treated group showed no significant changes (**Figures 6E, F**). Moreover, RNA pull-down assays also showed that the CASC2-wt probe pulled down EIF4A3 in GSC2C and GSC4D, rather than the CASC2-mt probe (**Figures 6G, H**).

We also performed RT-qPCR and found that the expression of CASC2 increased in GSC4D after EIF4A3 knockdown while decreased in GSC2C after EIF4A3 overexpression (**Figure 6I**). To further elucidate the mechanism of EIF4A3 downregulated CASC2 expression, we next analyzed the transcription of nascent CASC2 in EIF4A3-silenced GSC4D and EIF4A3-overexpressed GSC2C by RT-qPCR. All these results showed that EIF4A3 could not regulate the transcription of nascent CASC2 (**Figure 6J**). However, RNA stability measurement showed that the half-life of CASC2 was significantly prolonged after EIF4A3 knockdown, while shortened after EIF4A3 overexpression (**Figure 6K**). Taken together, these results suggested that EIF4A3 could bind to CASC2 and downregulated the expression of CASC2 *via* inducing its cleavage.

### RORA Transcriptionally Regulates the Expression of EIF4A3

We further explored the interaction between RORA and EIF4A3. Both RT-qPCR and western blotting showed that the expressions of EIF4A3 were increased after RORA knockdown while decreased after RORA overexpression (**Figures 6L, M**). We next performed luciferase reporter assays and found that the relative luciferase activities of EIF4A3 significantly increased in RORA-silenced GSC2C. However, the opposite results were obtained in RORA-overexpressed GSC4D (**Figure 6N**). All these results suggested that RORA transcriptionally inhibited the expression of EIF4A3.

### EIF4A3 Induced the Migration, Invasion, and EMT of GBM, and These Induction Effects Were Restrained Following CASC2 Overexpression

To confirm the common effects of EIF4A3 and CASC2 on the migration, invasion, and EMT of GBM, we detected the migration, invasion, and EMT in EIF4A3-overexpressed GSC2C. All results showed that the migration rates, invasion rates, the relative number of mesenchymal-like cells, expression levels of vimentin and N-cadherin were significantly increased, and the relative number of epithelioid-like cells and E-cadherin expression were decreased in EIF4A3-overexpressed GSC2C, while the promoting effects of EIF4A3 overexpression were reversed after CASC2 overexpression (**Figures S7A–D**). In summary, EIF4A3 promoted the migration, invasion, and EMT of GBM, and the promoting effects were reversed following CASC2 overexpression. Besides, we detected the expression of TGF-β1 after CASC2 or EIF4A3 knockdown or overexpression by qPCR and western blotting. The results showed that CASC2 negatively regulated TGF-β1 expression (**Figures S8A–D**), while EIF4A3 positively regulated the expression of TGF-β1 (**Figures S8E–H**).

### The EIF4A3/CASC2/RORA Feedback Loop Regulates GBM Tumorigenesis *In Vivo*

**Figure 7:**
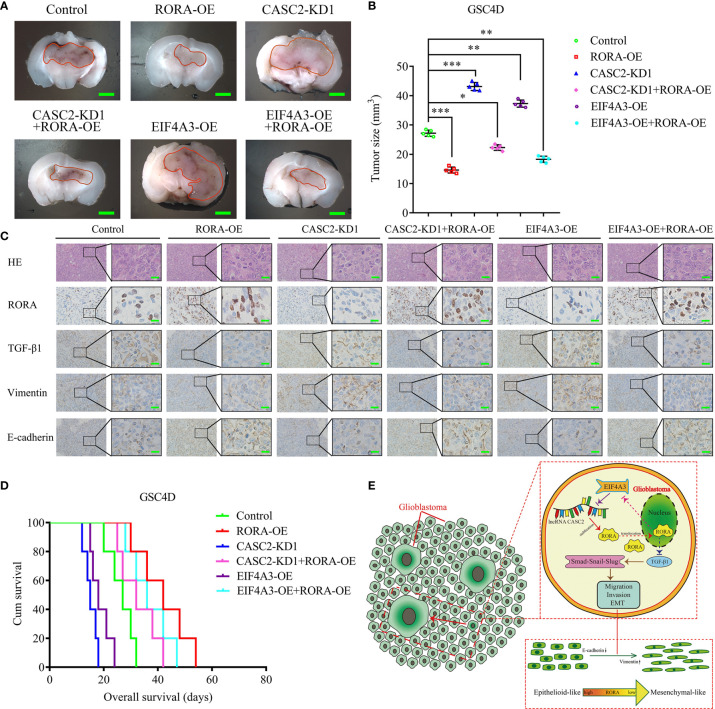
The EIF4A3/CASC2/RORA axis regulates GBM tumorigenesis *in vivo*. **(A)** Representative image shows the size of intracranial tumors in the coronal location of six groups (negative control, RORA overexpression, CASC2 knockdown, CASC2 knockdown combined with RORA overexpression, EIF4A3 overexpression, and EIF4A3 overexpression combined with RORA overexpression in GSC4D). Scale bar = 10 mm. **(B)** The measured tumor volumes among six groups are indicated. **(C)** Representative immunohistochemical staining showing the changes in RORA, TGF-β1, vimentin, and E-cadherin in the negative control, RORA overexpression, CASC2 knockdown, CASC2 knockdown combined with RORA overexpression, EIF4A3 overexpression, and EIF4A3 overexpression combined with RORA overexpression in orthotopic xenograft models. Scale bar = 50 μm. **(D)** Kaplan-Meier survival curves show that the CASC2 knockdown and EIF4A3 overexpression in GSC4D shortened the survival times of nude mice, while it prolonged the survival times after RORA overexpression, CASC2 knockdown combined with RORA overexpression, and EIF4A3 overexpression combined with RORA overexpression. For each group, n = 5. **(E)** Schematic diagram showing that the EIF4A3/CASC2/RORA axis regulated the migration, invasion, and EMT of GBMs through the TGF-β1/Smad signaling pathway. EV, empty vector; OE, overexpression; NC, negative control; KD, knockdown. All data are expressed as the mean ± SD (three independent experiments). ^*^P < 0.05; ^**^P < 0.01; ^***^P < 0.001.

To evaluate the effects of RORA on GBM tumorigenesis, we further constructed orthotopic xenograft models. The results showed that tumor volumes were decreased in the RORA-OE group, the CASC2-KD1+RORA-OE group, and the EIF4A3-OE+RORA-OE group when compared with the control group, while the tumor volumes were significantly increased in the CASC2-KD1 group and the EIF4A3-OE group compared with the control group (**Figures 7A, B**). Besides, IHC assays showed that in the RORA-OE group, the CASC2-KD1+RORA-OE group, and the EIF4A3-OE+RORA-OE group, the staining intensity and expression levels of RORA and E-cadherin were increased compared with the control group, while the expression levels of TGF-β1 and vimentin were decreased. However, the opposite results were obtained in the CASC2-KD1 group and the EIF4A3-OE group (**Figure 7C**). Moreover, Kaplan-Meier survival analysis showed that the survival times in the RORA-OE group, the CASC2-KD1+RORA-OE group, and the EIF4A3-OE+RORA-OE group were longer than the control group, while the opposite results were obtained in the CASC2-KD1 group and the EIF4A3-OE group (**Figure 7D**). To illustrate our findings, the schematic diagram in **Figure 7E** shows that the EIF4A3/CASC2/RORA feedback loop regulates the tumorigenesis, migration, invasion, and EMT of GBM through negatively affecting the TGF-β1/Smad signaling pathway.

## Discussion

Glioblastoma is the most frequent and aggressive primary brain tumor associated with a poor prognosis (31). In our previous study, RORA was confirmed as a suppressor in glioma, which inhibited the proliferation and tumorigenesis of glioma cell lines and GSCs (9). In the present study, we furtherly found that RORA expression in GBM was lower than in lower-grade glioma, indicating that RORA may inhibit the invasion and migration in GBMs. Studies have shown that EMT is an important cause of enhanced GBM invasiveness, tolerance to radiotherapy and chemotherapy, and tumor recurrence (8, 32). We further confirmed that RORA inhibited the migration, invasion, and EMT of GBMs.

In addition, we characterized the possible downstream mechanism involved in RORA inhibition of migration, invasion, and EMT of GBMs. We conducted a GSEA based on CGGA and TCGA GBM databases and found that the TGF-β signaling pathway was enriched in the higher RORA expression groups. However, all RT-qPCR, western blotting, and ELISA results showed that RORA negatively regulated TGF-β1 expression. Moreover, several studies have confirmed that the TGF-β1/Smad signaling pathway is involved in the invasion and EMT of GBM (10, 33, 34). We further confirmed that RORA inhibited the migration, invasion, and EMT of GBMs by negatively regulating the TGF-β1/Smad signaling pathway.

It has been reported that lncRNAs play vital roles in regulating the biological processes of tumors, including GBM (35). For example, LncRNA PVT1 promotes tumorigenesis and glioma progression by regulating the miR-128-3p/GREM1 axis and BMP signaling pathway (36). LncRNA PLAC2 downregulates the expression of RPL36 and blocks cell cycle progression in glioma *via* a mechanism involving STAT1 (37). In the present study, it was predicted that CASC2 could bind to RORA proteins according to catRAPID. Previous studies have already reported the low expression of CASC2 in glioma, where it acts as a tumor suppressor, resulting in a poorer prognosis and clinicopathological features of glioma patients (28, 29, 38). Our study further confirmed that CASC2 could bind to and maintain the stability of RORA protein and also promote the nuclear translocation of RORA. Besides, we certified that CASC2 inhibited the migration, invasion, and EMT of GBM, and the inhibitory effects could be reversed by RORA knockdown. All of these results suggested that there existed an interaction between CASC2 and RORA.

Previous studies illustrated that RBPs could bind to RNAs and regulate their transcription, splicing, editing, translocation, and stability (30, 39). Since EIF4A3 is involved in various RNA metabolic processes, including nonsense-mediated RNA decay and RNA splicing, for example, EIF4A3-induced circular RNA ASAP1 (circASAP1) facilitates tumorigenesis and temozolomide resistance of glioblastoma *via* NRAS/MEK1/ERK1/2 signaling pathway (40). In the present study, it was predicted that EIF4A3 was the most probable candidate RBP to interact with CASC2 according to the Starbase database. We further confirmed that EIF4A3 could bind to CASC2 *via* RIP assay and RNA pull-down assay. Moreover, we found that EIF4A3 downregulated the expression of CASC2 *via* inducing its cleavage. We further explored the interaction between RORA and EIF4A3 and found that RORA transcriptionally inhibited the expression of EIF4A3. *In vitro* experiments, we confirmed that EIF4A3 induced the migration, invasion, and EMT of GBM, and these induction effects were restrained following CASC2 overexpression. So far, we conclude that the EIF4A3/CASC2/RORA feedback loop is involved in the migration, invasion, and EMT of GBMs. Finally, we confirmed that the EIF4A3/CASC2/RORA feedback loop participated in the tumorigenesis *in vivo*.

In summary, we first found abnormally low expression of RORA in GBM in clinical specimens. Then gene set enrichment analysis (GSEA) and *in vivo* and *in vitro* experiments showed that RORA might inhibit the migration, invasion, and EMT of GBM by regulating the TGF-β1/Smad signaling pathway. Mechanistically, we found EIF4A3 could downregulate the expression of CASC2 *via* inducing its cleavage. Besides, CASC2 can bind to, maintain the stability, and promote the nuclear translocation of RORA protein. RORA can furtherly transcriptionally inhibit the expression of EIF4A3, which forms a feedback loop among EIF4A3/CASC2/RORA. Therefore, the lower expression of RORA played a vital role in the invasiveness of GBMs *via* EIF4A3/CASC2/RORA feedback loop regulated TGF-β1/Smad signaling pathway might represent a promising therapeutic strategy for the treatment of GBM.

## Data Availability Statement

The original contributions presented in the study are included in the article/**Supplementary Material**. Further inquiries can be directed to the corresponding author.

## Ethics Statement

The studies involving human participants were reviewed and approved by the First Hospital of China Medical University research ethics committee. The patients/participants provided their written informed consent to participate in this study. The animal study was reviewed and approved by the First Hospital of China Medical University research ethics committee.

## Author Contributions

ZTJ conceived and designed the study. JSZ, YJ, and LC performed the experiments, HL collected the data. HYZ, JPZ, and YM performed bioinformatics analysis and analyzed the data. JSZ and YJ interpreted results and wrote the manuscript. JSZ, YJ, and LC contributed equally to this work. All authors contributed to the article and approved the submitted version.

## Funding

This work was supported by the National Natural Science Foundation of China (Nos. 82072794), the Major Disease Prevention and Control Technology Action Plan of China (2018ZX-07S-006), the Liaoning BaiQianWan Talents Program (No. 2019-B45), the Social Development Program from Shenyang Science and Technology Bureau, China (20-205-4-075), the Project of JiangSu XianSheng Pharmaceutical Co. (JSXSZD-SA2020-06-004), China Postdoctoral Science Foundation (No. 267285), and the Shanghai Sailing Program (No. 21YF1449900). The funder, JiangSu XianSheng Pharmaceutical Co., was not involved in the study design, collection, analysis, and interpretation of data, the writing of this article or the decision to submit it for publication.

## Conflict of Interest

The authors declare that the research was conducted in the absence of any commercial or financial relationships that could be construed as a potential conflict of interest.

## Publisher’s Note

All claims expressed in this article are solely those of the authors and do not necessarily represent those of their affiliated organizations, or those of the publisher, the editors and the reviewers. Any product that may be evaluated in this article, or claim that may be made by its manufacturer, is not guaranteed or endorsed by the publisher.

## Supplementary Material

Supplementary Figure 1 The validation of glioma stem cells. **(A)** Representative immunofluorescence staining of CD133 and nestin in patient-derived GSCs. Scale bar = 50 μm. **(B)** The patient-derived GSCs became adherent and differentiated into GFAP or β III tubulin-positive cells. Scale bar = 50 μm.

Supplementary Figure 2 RORA inhibits the migration, invasion, and EMT of GBM *in vitro*. **(A, B)** qPCR **(A)** and western blotting **(B)** showing the validation of RORA knockdown. **(C, D)** Representative migration assay and transwell assay showing the migration rates and invasion rates of U87 cells and GSC2C with RORA knockdown, and the negative control. Scale bar = 100 μm. **(E)** Representative microphotographs showing the morphological changes in RORA-silenced U87 cells and GSC2C. Scale bar = 50 μm. **(F, G)** Representative immunofluorescence staining **(F)** and western blotting **(G)** showing the changes in E-cadherin, vimentin, and N-cadherin in U87 cells and GSC2C after RORA knockdown. Scale bar = 50 μm. NC, negative control; KD, knockdown. All data are expressed as the mean ± SD (three independent experiments). ^*^P < 0.05; ^**^P < 0.01; ^***^P < 0.001.

Supplementary Figure 3 Western blotting showing the expression of non-canonical TGF-β/Smad signaling, including MAPK pathways, JNK, p38, and PI3K cascade after RORA overexpression. EV, empty vector; OE, overexpression.

Supplementary Figure 4 RORA regulates the migration, invasion, and EMT of GBM by negatively affecting the TGF-β1/Smad signaling pathway. **(A, B)** Representative migration assays and Transwell assays showing the migration rates and invasion rates of RORA-overexpressed GSC4D after TGF-β1 treatment, and the negative control treated with dimethyl sulfoxide. Scale bar = 100 μm. **(C)** Representative microphotographs showing the morphological changes in RORA-overexpressed GSC4D after TGF-β1 treatment. Scale bar = 50 μm. **(D, E)** Representative western blotting **(D)** and immunofluorescence staining **(E)** showing the changes in E-cadherin, vimentin, N-cadherin, and the downstream targets of the TGF-β1/Smad signaling pathway in RORA-overexpressed GSC4D after TGF-β1 treatment. Scale bar = 50 μm. EV, empty vector; OE, overexpression. All data are expressed as the mean ± SD (three independent experiments). ^*^P < 0.05; ^**^P < 0.01; ^***^P < 0.001.

Supplementary Figure 5 RORA regulates the proliferation of GBM by negatively affecting the TGF-β1/Smad signaling pathway. **(A)** MTS assay showing the cell viability decreased in T98G cells after RORA overexpression, while increased following TGF-β1 treatment. **(B)** EDU assay showing the relative ratio of EDU positive cells decreased in T98G cells after RORA overexpression, while increased following TGF-β1 treatment. Scale bar = 100 μm. EV, empty vector; OE, overexpression. All data are expressed as the mean ± SD (three independent experiments). *P < 0.05; **P < 0.01; ***P < 0.001.

Supplementary Figure 6 CASC2 regulates the expression of RORA protein rather than the expression of RORA mRNA. **(A, B)** Western blotting showing RORA protein expression after CASC2 overexpression **(A)** or knockdown **(B)**. **(C, D)** RT-qPCR showing the expression of RORA mRNA after CASC2 overexpression **(C)** or knockdown **(D)**. EV, empty vector; OE, overexpression; NC, negative control; KD, knockdown. All data are expressed as the mean ± SD (three independent experiments). ^*^P < 0.05; ^**^P < 0.01; ^***^P < 0.001.

Supplementary Figure 7 EIF4A3 induced the migration, invasion, and EMT of GBM, and these induction effects were restrained following CASC2 overexpression. **(A, B)** Representative migration assay and Transwell assay showing the migration rates and invasion rates of GSC2C with EIF4A3 overexpression, while reversed after CASC2 overexpression. Scale bar = 100 μm. Representative microphotographs showing the morphological changes in EIF4A3-overexpressed GSC2C, while also reversed after CASC2 overexpression. Scale bar = 50 μm. **(C, D)** Representative immunofluorescence staining and western blotting showing the changes in E-cadherin, vimentin, and N-cadherin in GSC2C after EIF4A3 overexpression, while reversed after CASC2 overexpression. Scale bar = 50 μm. EV, empty vector; OE, overexpression; NC, negative control; KD, knockdown. All data are expressed as the mean ± SD (three independent experiments). ^*^P < 0.05; ^**^P < 0.01; ^***^P < 0.001.

Supplementary Figure 8 The expression of TGF-β1 after CASC2 or EIF4A3 knockdown or overexpression. **(A, B)** qPCR **(A)** and western blotting **(B)** showing the expression of TGF-β1 increased in U87 cells and GSC2C after CASC2 knockdown. **(C, D)** qPCR **(C)** and western blotting **(D)** showing the expression of TGF-β1 decreased in T98G cells and GSC4D after CASC2 overexpression. **(E, F)** qPCR **(E)** and western blotting **(F)** showing the expression of TGF-β1 decreased in U87 cells and GSC2C after EIF4A3 knockdown. **(G, H)** qPCR **(G)** and western blotting **(H)** showing the expression of TGF-β1 increased in T98G cells and GSC4D after EIF4A3 overexpression. EV, empty vector; OE, overexpression; NC, negative control; KD, knockdown. All data are expressed as the mean ± SD (three independent experiments). *P < 0.05; **P < 0.01; ***P < 0.001.

## Abbreviations

EMT, Epithelial-mesenchymal transition; GBM, glioblastoma; GSCs, glioma stem cells; RORA, Retinoic acid receptor-related orphan receptor A; lncRNAs, Long non-coding RNAs; RBPs, RNA-binding proteins; Eukaryotic initiation factor 4A3: EIF4A3; GSEA, Gene set enrichment analysis; RT-qPCR, Real-Time Quantitative Reverse Transcription PCR; IHC, Immunohistochemistry; ELISA, Enzyme-linked immunosorbent assay; RIP, RNA immunoprecipitation; CGGA, Chinese Glioma Genome Atlas; TCGA, The Cancer Genome Atlas; RISC, RNA-induced silencing complex; LGG, lower-grade glioma; EV, Empty vector; OE, Overexpression; NC, Negative control; KD, Knockdown.

## References

[1] Delgado-Lopez PD Corrales-Garcia EM. Survival in Glioblastoma: A Review on the Impact of Treatment Modalities. Clin Transl Oncol (2016) 18(11):1062–71. 10.1007/s12094-016-1497-x 26960561

[2] Braun K Ahluwalia MS. Treatment of Glioblastoma in Older Adults. Curr Oncol Rep (2017) 19(12):81. 10.1007/s11912-017-0644-z 29075865

[3] Sotoudeh H Shafaat O Bernstock JD Brooks MD Elsayed GA Chen JA. Artificial Intelligence in the Management of Glioma: Era of Personalized Medicine. Front Oncol (2019) 9:768. 10.3389/fonc.2019.00768 31475111 PMC6702305

[4] Scott JG Bauchet L Fraum TJ Nayak L Cooper AR Chao ST. Recursive Partitioning Analysis of Prognostic Factors for Glioblastoma Patients Aged 70 Years or Older. Cancer (2012) 118(22):5595–600. 10.1002/cncr.27570 PMC3402652 22517216

[5] Rajesh Y Pal I Banik P Chakraborty S Borkar SA Dey G. Insights Into Molecular Therapy of Glioma: Current Challenges and Next Generation Blueprint. Acta Pharmacol Sin (2017) 38(5):591–613. 10.1038/aps.2016.167 28317871 PMC5457688

[6] Jetten AM. Retinoid-Related Orphan Receptors (RORs): Critical Roles in Development, Immunity, Circadian Rhythm, and Cellular Metabolism. Nucl Receptor Signaling (2009) 7:e003. 10.1621/nrs.07003 PMC2670432 19381306

[7] Lu Y Yi Y Liu P Wen W James M Wang D. Common Human Cancer Genes Discovered by Integrated Gene-Expression Analysis. PloS One (2007) 2(11):e1149. 10.1371/journal.pone.0001149 17989776 PMC2065803

[8] Bhat KPL Balasubramaniyan V Vaillant B Ezhilarasan R Hummelink K Hollingsworth F. Mesenchymal Differentiation Mediated by NF-κb Promotes Radiation Resistance in Glioblastoma. Cancer Cell (2013) 24(3):331–46. 10.1016/j.ccr.2013.08.001 PMC3817560 23993863

[9] Jiang Y Zhou J Zhao J Hou D Zhang H Li L. MiR-18a-Downregulated RORA Inhibits the Proliferation and Tumorigenesis of Glioma Using the TNF-Alpha-Mediated NF-kappaB Signaling Pathway. EBioMedicine (2020) 52:102651. 10.1016/j.ebiom.2020.102651 32062354 PMC7016377

[10] Jiang Y Zhou J Hou D Luo P Gao H Ma Y. Prosaposin is a Biomarker of Mesenchymal Glioblastoma and Regulates Mesenchymal Transition Through the TGF-β1/Smad Signaling Pathway. J Pathol (2019) 249(1):26–38. 10.1002/path.5278 30953361

[11] Guo Z Li G Bian E Ma CC Wan J Zhao B. TGF-β-Mediated Repression of MST1 by DNMT1 Promotes Glioma Malignancy. BioMed Pharmacother (2017) 94:774–80. 10.1016/j.biopha.2017.07.081 28802229

[12] Wang Q Cai J Fang C Yang C Zhou J Tan Y. Mesenchymal Glioblastoma Constitutes a Major ceRNA Signature in the TGF-β Pathway. Theranostics (2018) 8(17):4733–49. 10.7150/thno.26550 PMC6160778 30279734

[13] Zhang XQ Leung GK. Long non-Coding RNAs in Glioma: Functional Roles and Clinical Perspectives. Neurochem Int (2014) 77:78–85. 10.1016/j.neuint.2014.05.008 24887176

[14] Chen M Wu X Ma W Zhou Q Wang X Zhang R. Decreased Expression of lncRNA VPS9D1-AS1 in Gastric Cancer and its Clinical Significance. Cancer Biomark (2017) 21(1):23–8. 10.3233/CBM-170172 PMC13075736 29036784

[15] Cech TR Steitz JA. The Noncoding RNA Revolution-Trashing Old Rules to Forge New Ones. Cell (2014) 157(1):77–94. 10.1016/j.cell.2014.03.008 24679528

[16] Yao Y Ma J Xue Y Wang P Li Z Liu J. Knockdown of Long Non-Coding RNA XIST Exerts Tumor-Suppressive Functions in Human Glioblastoma Stem Cells by Up-Regulating miR-152. Cancer Lett (2015) 359(1):75–86. 10.1016/j.canlet.2014.12.051 25578780

[17] Baldinu P Cossu A Manca A Satta MP Sini MC Rozzo C. Identification of a Novel Candidate Gene, CASC2, in a Region of Common Allelic Loss at Chromosome 10q26 in Human Endometrial Cancer. Hum Mutat (2004) 23(4):318–26. 10.1002/humu.20015 15024726

[18] Wang P Liu YH Yao YL Li Z Li ZQ Ma J. Long Non-Coding RNA CASC2 Suppresses Malignancy in Human Gliomas by miR-21. Cell Signal (2015) 27(2):275–82. 10.1016/j.cellsig.2014.11.011 25446261

[19] Gerstberger S Hafner M Tuschl T. A Census of Human RNA-Binding Proteins. Nat Rev Genet (2014) 15(12):829–45. 10.1038/nrg3813 PMC11148870 25365966

[20] Ito M Tanaka T Cary DR Iwatani-Yoshihara M Kamada Y Kawamoto T. Discovery of Novel 1,4-Diacylpiperazines as Selective and Cell-Active Eif4a3 Inhibitors. J Med Chem (2017) 60(8):3335–51. 10.1021/acs.jmedchem.6b01904 28358513

[21] Chan CC Dostie J Diem MD Feng W Mann M Rappsilber J. Eif4a3 is a Novel Component of the Exon Junction Complex. RNA (2004) 10(2):200–9. 10.1261/rna.5230104 PMC1370532 14730019

[22] Tang W Wang D Shao L Liu X Zheng J Xue Y. LINC00680 and TTN-AS1 Stabilized by EIF4A3 Promoted Malignant Biological Behaviors of Glioblastoma Cells. Mol Ther Nucleic Acids (2020) 19:905–21. 10.1016/j.omtn.2019.10.043 PMC7063483 32000032

[23] Jiang Y Han S Cheng W Wang Z Wu A. NFAT1-Regulated IL6 Signalling Contributes to Aggressive Phenotypes of Glioma. Cell Commun Signal: CCS (2017) 15(1):54. 10.1186/s12964-017-0210-1 29258522 PMC5735798

[24] Jiang Y Zhou J Zhao J Zhang H Li L Li H. The U2AF2/circRNA ARF1/miR-342-3p/ISL2 Feedback Loop Regulates Angiogenesis in Glioma Stem Cells. J Exp Clin Cancer Res (2020) 39(1):182. 10.1186/s13046-020-01691-y 32894165 PMC7487667

[25] Jiang Y Song Y Wang R Hu T Zhang D Wang Z. NFAT1-Mediated Regulation of NDEL1 Promotes Growth and Invasion of Glioma Stem-Like Cells. Cancer Res (2019) 79(10):2593–603. 10.1158/0008-5472.CAN-18-3297 30940662

[26] Batista PJ Chang HY. Long Noncoding RNAs: Cellular Address Codes in Development and Disease. Cell (2013) 152(6):1298–307. 10.1016/j.cell.2013.02.012 PMC3651923 23498938

[27] Hua Q Mi B Xu F Wen J Zhao L Liu J. Hypoxia-Induced lncRNA-AC020978 Promotes Proliferation and Glycolytic Metabolism of Non-Small Cell Lung Cancer by Regulating PKM2/HIF-1alpha Axis. Theranostics (2020) 10(11):4762–78. 10.7150/thno.43839 PMC7163453 32308748

[28] Liao Y Shen L Zhao H Liu Q Fu J Guo Y. LncRNA CASC2 Interacts With miR-181a to Modulate Glioma Growth and Resistance to TMZ Through PTEN Pathway. J Cell Biochem (2017) 118(7):1889–99. 10.1002/jcb.25910 28121023

[29] Wang R Li Y Zhu G Tian B Zeng W Yang Y. Long Noncoding RNA CASC2 Predicts the Prognosis of Glioma Patients and Functions as a Suppressor for Gliomas by Suppressing Wnt/β-Catenin Signaling Pathway. Neuropsychiatr Dis Treat (2017) 13:1805–13. 10.2147/NDT.S137171 PMC5513825 28744130

[30] Zhang F Ruan X Ma J Liu X Zheng J Liu Y. DGCR8/ZFAT-AS1 Promotes CDX2 Transcription in a PRC2 Complex-Dependent Manner to Facilitate the Malignant Biological Behavior of Glioma Cells. Mol Ther (2020) 28(2):613–30. 10.1016/j.ymthe.2019.11.015 PMC7001006 31813799

[31] Georgescu MM Olar A. Genetic and Histologic Spatiotemporal Evolution of Recurrent, Multifocal, Multicentric and Metastatic Glioblastoma. Acta Neuropathol Commun (2020) 8(1):10. 10.1186/s40478-020-0889-x 32014051 PMC6998196

[32] Zheng X Carstens JL Kim J Scheible M Kaye J Sugimoto H. Epithelial-To-Mesenchymal Transition is Dispensable for Metastasis But Induces Chemoresistance in Pancreatic Cancer. Nature (2015) 527(7579):525–30. 10.1038/nature16064 PMC4849281 26560028

[33] Srivastava C Irshad K Dikshit B Chattopadhyay P Sarkar C Gupta DK. FAT1 Modulates EMT and Stemness Genes Expression in Hypoxic Glioblastoma. Int J Cancer (2018) 142(4):805–12. 10.1002/ijc.31092 28994107

[34] Kesanakurti D Maddirela D Banasavadi-Siddegowda YK Lai TH Qamri Z Jacob NK. A Novel Interaction of PAK4 With Pparγ to Regulate Nox1 and Radiation-Induced Epithelial-to-Mesenchymal Transition in Glioma. Oncogene (2017) 36(37):5309–20. 10.1038/onc.2016.261 PMC5599308 28534509

[35] Yang G Lu X Yuan L. LncRNA: A Link Between RNA and Cancer. Biochim Biophys Acta (2014) 1839(11):1097–109. 10.1016/j.bbagrm.2014.08.012 25159663

[36] Fu C Li D Zhang X Liu N Chi G Jin X. LncRNA PVT1 Facilitates Tumorigenesis and Progression of Glioma *via* Regulation of MiR-128-3p/GREM1 Axis and BMP Signaling Pathway. Neurotherapeutics (2018) 15(4):1139–57. 10.1007/s13311-018-0649-9 PMC6277294 30120709

[37] Hu YW Kang CM Zhao JJ Nie Y Zheng L Li HX. LncRNA PLAC2 Down-Regulates RPL36 Expression and Blocks Cell Cycle Progression in Glioma Through a Mechanism Involving STAT1. J Cell Mol Med (2018) 22(1):497–510. 10.1111/jcmm.13338 28922548 PMC5742712

[38] Jiang C Shen F Du J Fang X Li X Su J. Upregulation of CASC2 Sensitized Glioma to Temozolomide Cytotoxicity Through Autophagy Inhibition by Sponging miR-193a-5p and Regulating mTOR Expression. BioMed Pharmacother (2018) 97:844–50. 10.1016/j.biopha.2017.10.146 29136760

[39] Müller-McNicoll M Neugebauer KM. How Cells Get the Message: Dynamic Assembly and Function of mRNA-Protein Complexes. Nat Rev Genet (2013) 14(4):275–87. 10.1038/nrg3434 23478349

[40] Wei Y Lu C Zhou P Zhao L Lyu X Yin J. EIF4A3-Induced Circular RNA ASAP1(circASAP1) Promotes Tumorigenesis and Temozolomide Resistance of Glioblastoma *via* NRAS/MEK1/ERK1/2 Signaling. Neuro Oncol (2021) 23(4):611–24. 10.1093/neuonc/noaa214 PMC8041353 32926734

