# Supplementary figures and images for: The EIF4A3/CASC2/RORA Feedback Loop Regulates the Aggressive Phenotype in Glioblastomas
Source: Front Oncol. 2021 Aug 2;11:699933. doi: 10.3389/fonc.2021.699933 (PMC8366401; doi:10.3389/fonc.2021.699933)

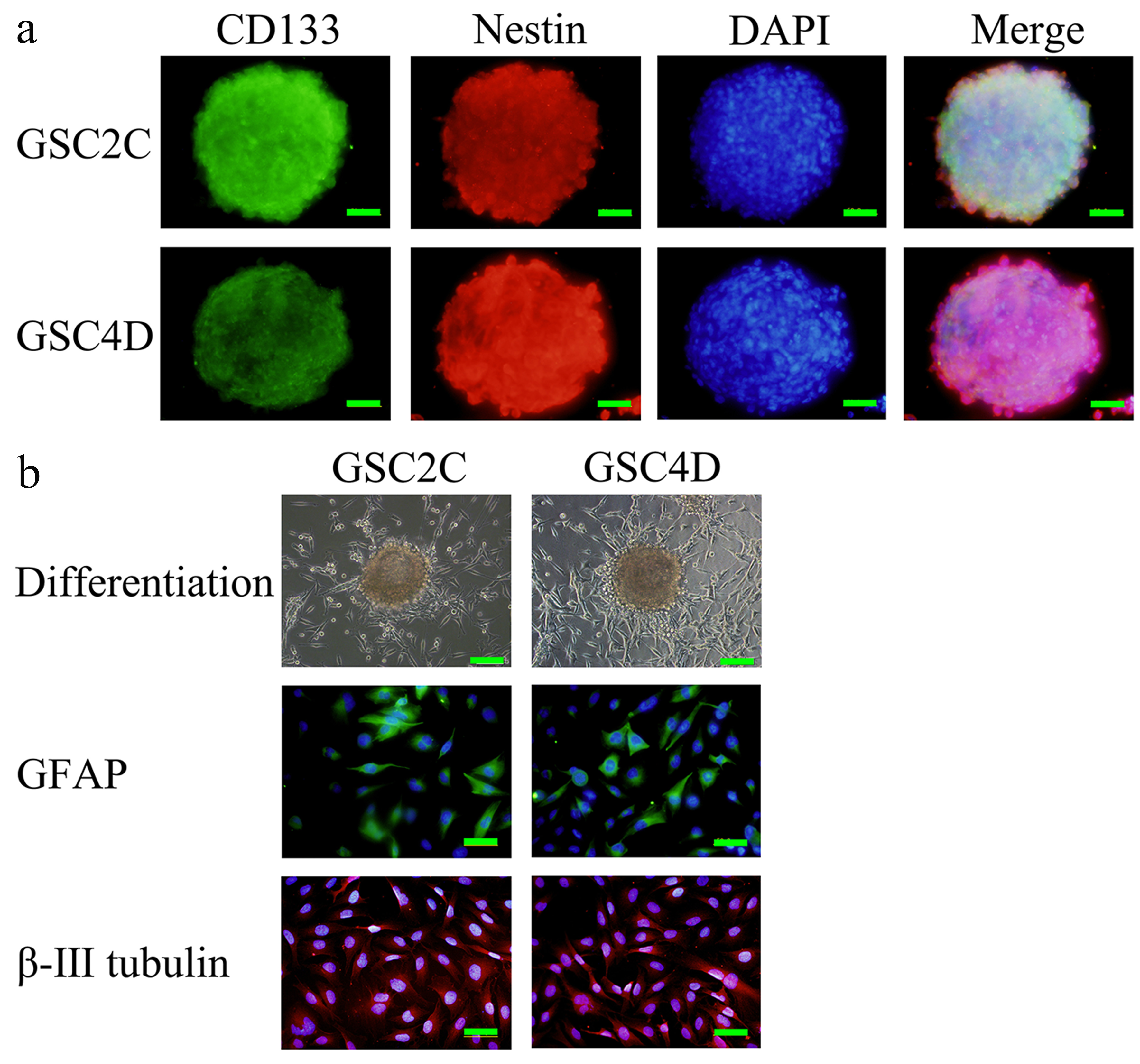

Supplement: Supplementary Figure 1 — The validation of glioma stem cells. (A) Representative immunofluorescence staining of CD133 and nestin in patient-derived GSCs. Scale bar = 50 μm. (B) The patient-derived GSCs became adherent and differentiated into GFAP or β III tubulin-positive cells. Scale bar = 50 μm. [file Image_1.tif]

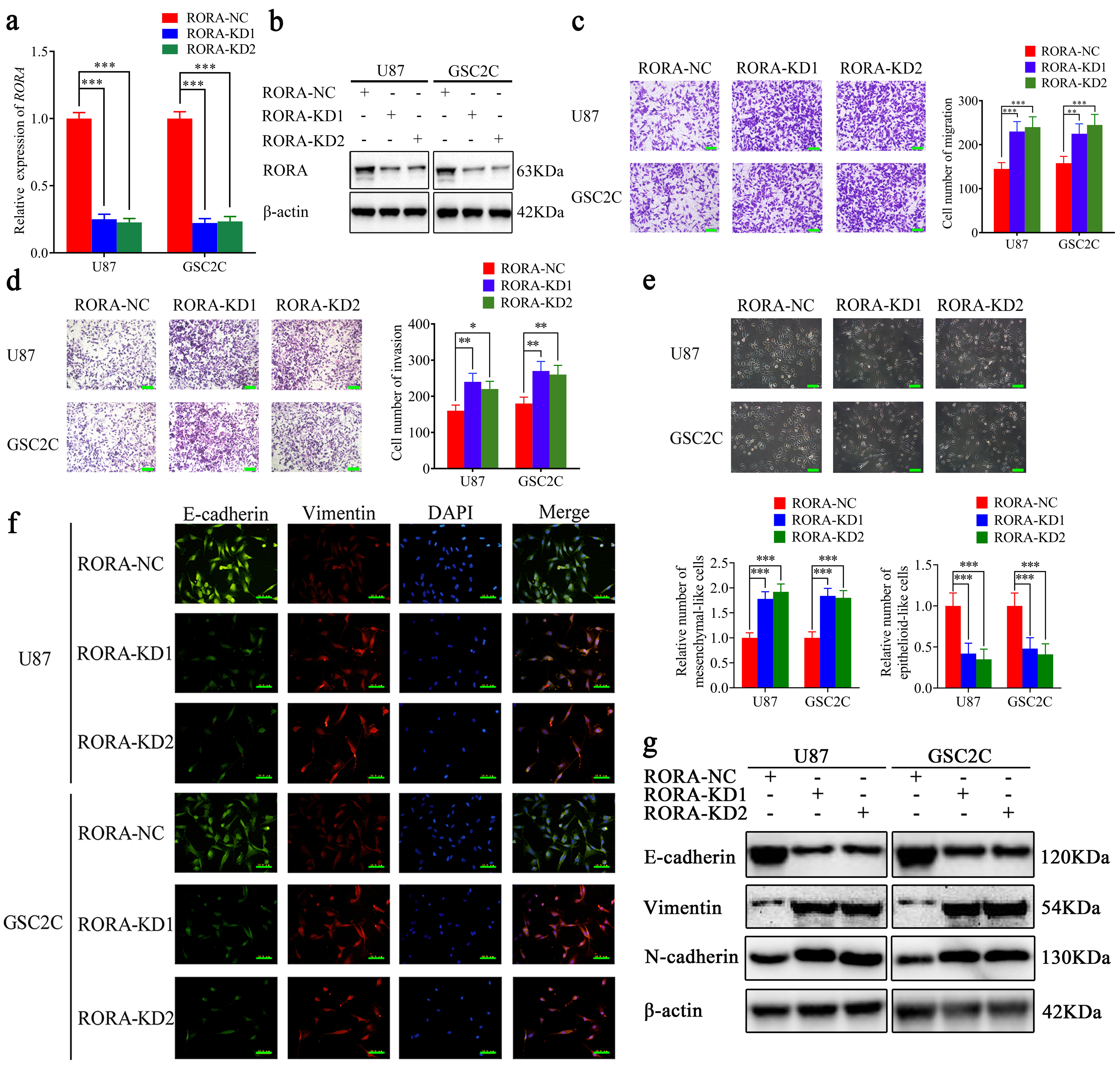

Supplement: Supplementary Figure 2 — RORA inhibits the migration, invasion, and EMT of GBM in vitro. (A, B) qPCR (A) and western blotting (B) showing the validation of RORA knockdown. (C, D) Representative migration assay and transwell assay showing the migration rates and invasion rates of U87 cells and GSC2C with RORA knockdown, and the negative control. Scale bar = 100 μm. (E) Representative microphotographs showing the morphological changes in RORA-silenced U87 cells and GSC2C. Scale bar = 50 μm. (F, G) Representative immunofluorescence staining (F) and western blotting (G) showing the changes in E-cadherin, vimentin, and N-cadherin in U87 cells and GSC2C after RORA knockdown. Scale bar = 50 μm. NC, negative control; KD, knockdown. All data are expressed as the mean ± SD (three independent experiments). *P < 0.05; **P < 0.01; ***P < 0.001. [file Image_2.tif]

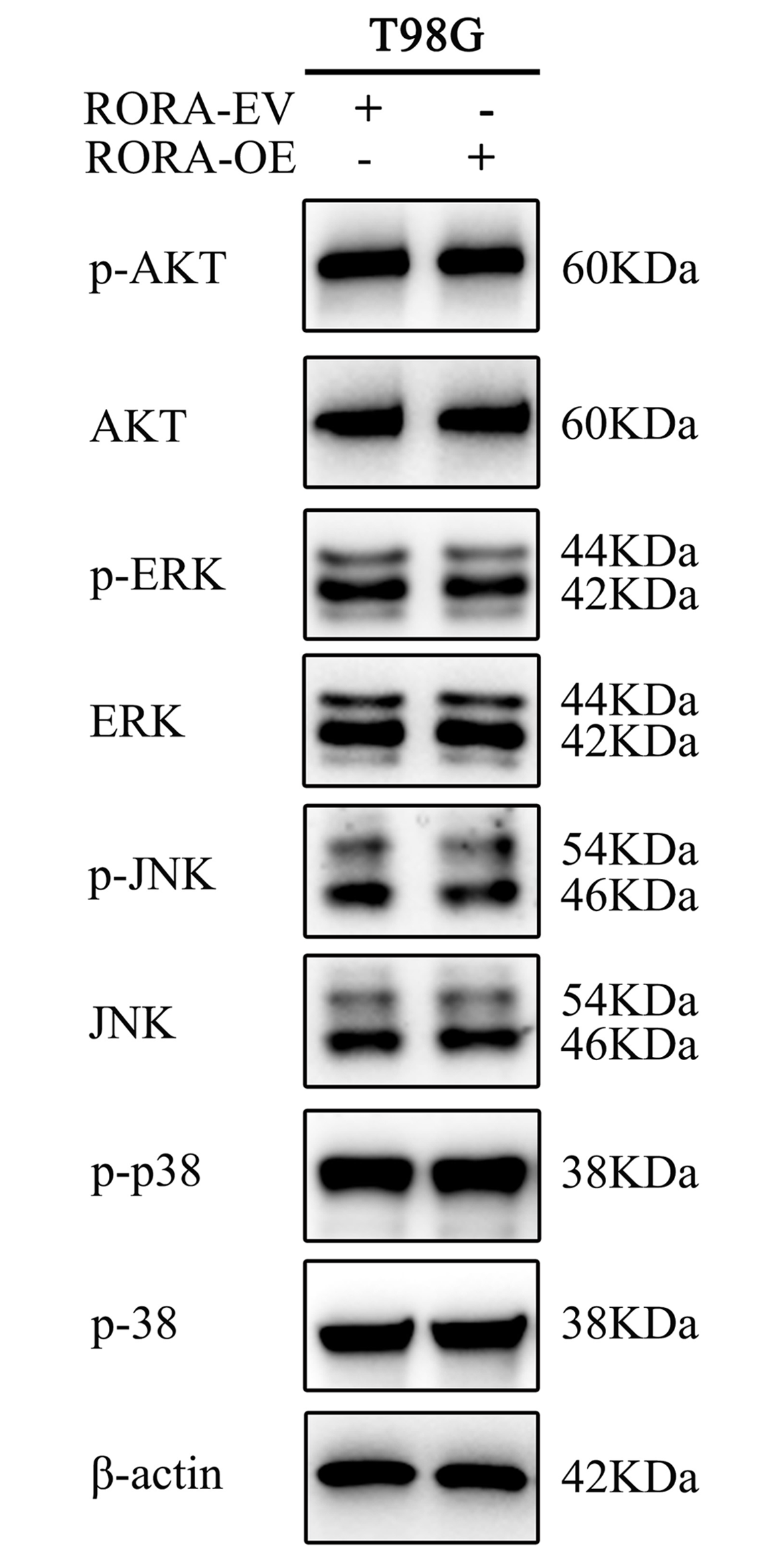

Supplement: Supplementary Figure 3 — Western blotting showing the expression of non-canonical TGF-β/Smad signaling, including MAPK pathways, JNK, p38, and PI3K cascade after RORA overexpression. EV, empty vector; OE, overexpression. [file Image_3.tif]

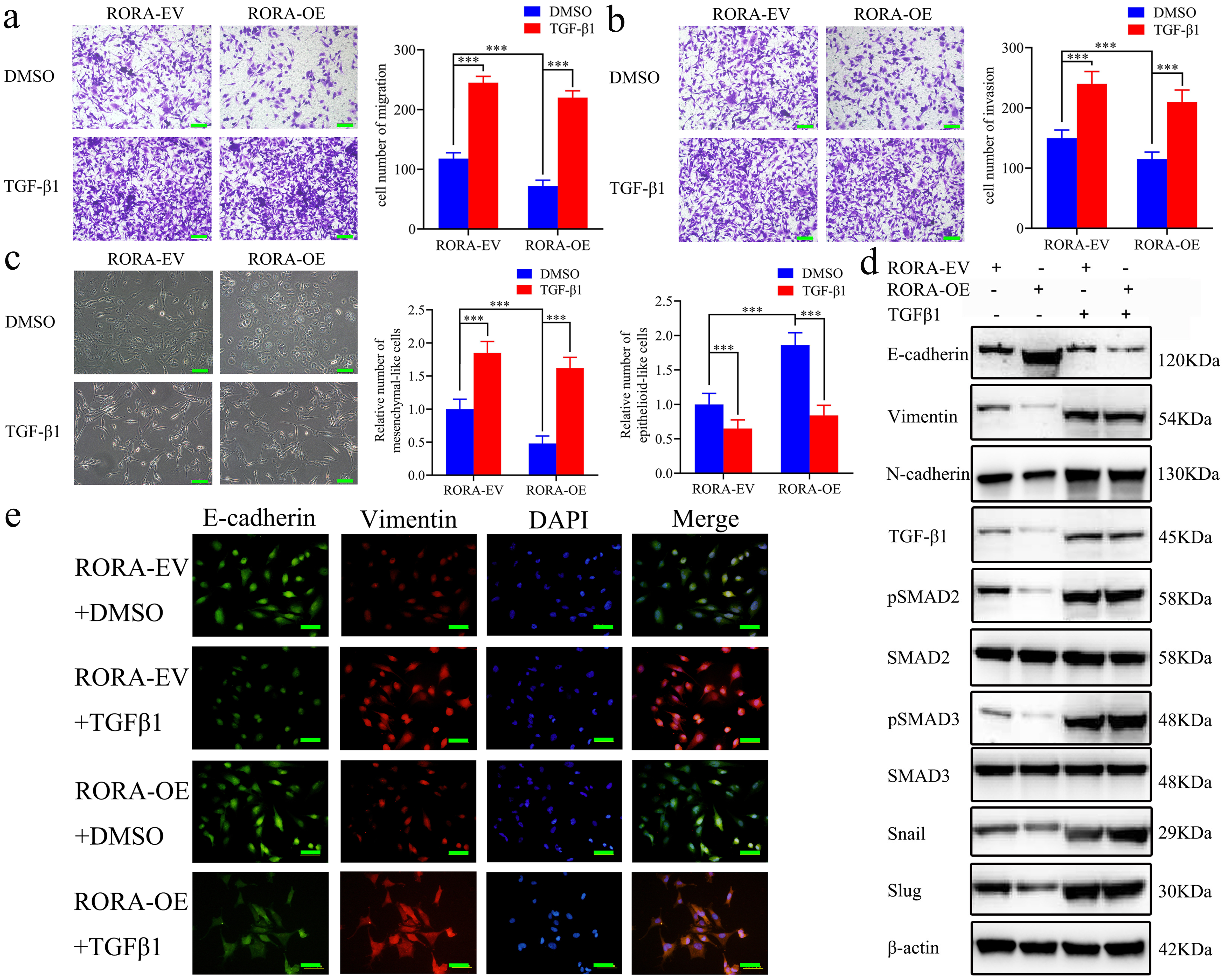

Supplement: Supplementary Figure 4 — RORA regulates the migration, invasion, and EMT of GBM by negatively affecting the TGF-β1/Smad signaling pathway. (A, B) Representative migration assays and Transwell assays showing the migration rates and invasion rates of RORA-overexpressed GSC4D after TGF-β1 treatment, and the negative control treated with dimethyl sulfoxide. Scale bar = 100 μm. (C) Representative microphotographs showing the morphological changes in RORA-overexpressed GSC4D after TGF-β1 treatment. Scale bar = 50 μm. (D, E) Representative western blotting (D) and immunofluorescence staining (E) showing the changes in E-cadherin, vimentin, N-cadherin, and the downstream targets of the TGF-β1/Smad signaling pathway in RORA-overexpressed GSC4D after TGF-β1 treatment. Scale bar = 50 μm. EV, empty vector; OE, overexpression. All data are expressed as the mean ± SD (three independent experiments). *P < 0.05; **P < 0.01; ***P < 0.001. [file Image_4.tif]

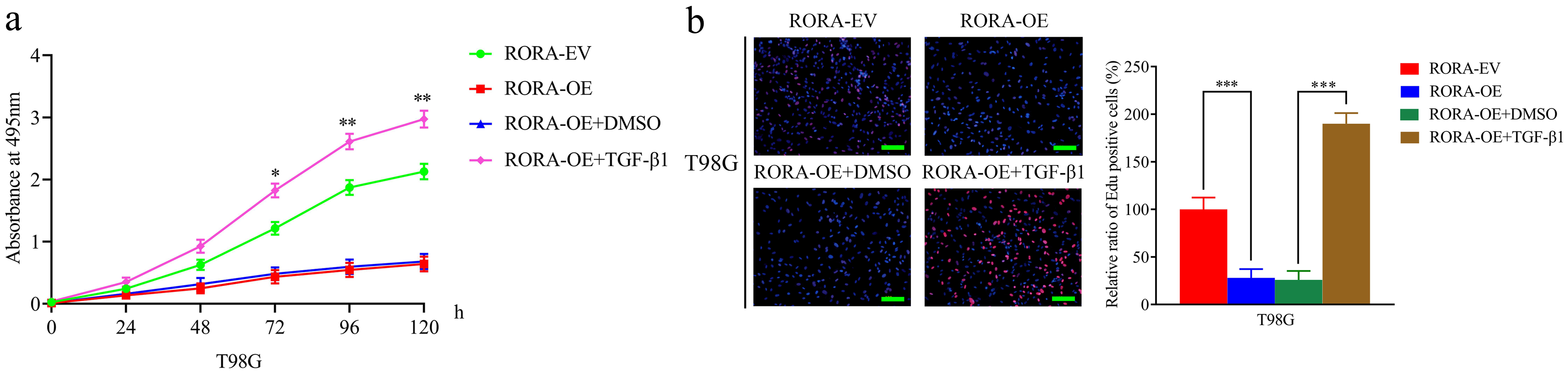

Supplement: Supplementary Figure 5 — RORA regulates the proliferation of GBM by negatively affecting the TGF-β1/Smad signaling pathway. (A) MTS assay showing the cell viability decreased in T98G cells after RORA overexpression, while increased following TGF-β1 treatment. (B) EDU assay showing the relative ratio of EDU positive cells decreased in T98G cells after RORA overexpression, while increased following TGF-β1 treatment. Scale bar = 100 μm. EV, empty vector; OE, overexpression. All data are expressed as the mean ± SD (three independent experiments). *P < 0.05; **P < 0.01; ***P < 0.001. [file Image_5.tif]

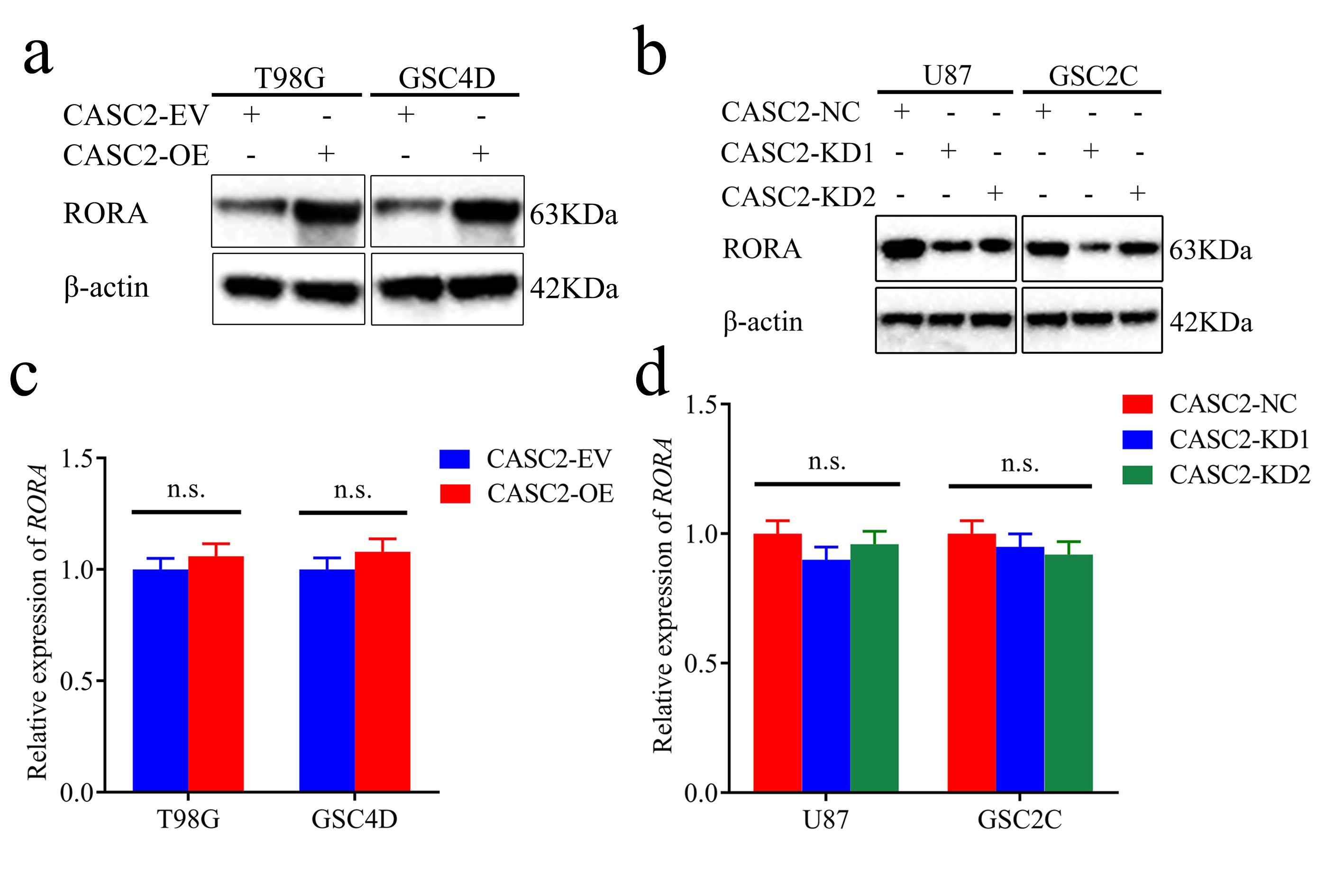

Supplement: Supplementary Figure 6 — CASC2 regulates the expression of RORA protein rather than the expression of RORA mRNA. (A, B) Western blotting showing RORA protein expression after CASC2 overexpression (A) or knockdown (B). (C, D) RT-qPCR showing the expression of RORA mRNA after CASC2 overexpression (C) or knockdown (D). EV, empty vector; OE, overexpression; NC, negative control; KD, knockdown. All data are expressed as the mean ± SD (three independent experiments). *P < 0.05; **P < 0.01; ***P < 0.001. [file Image_6.tif]

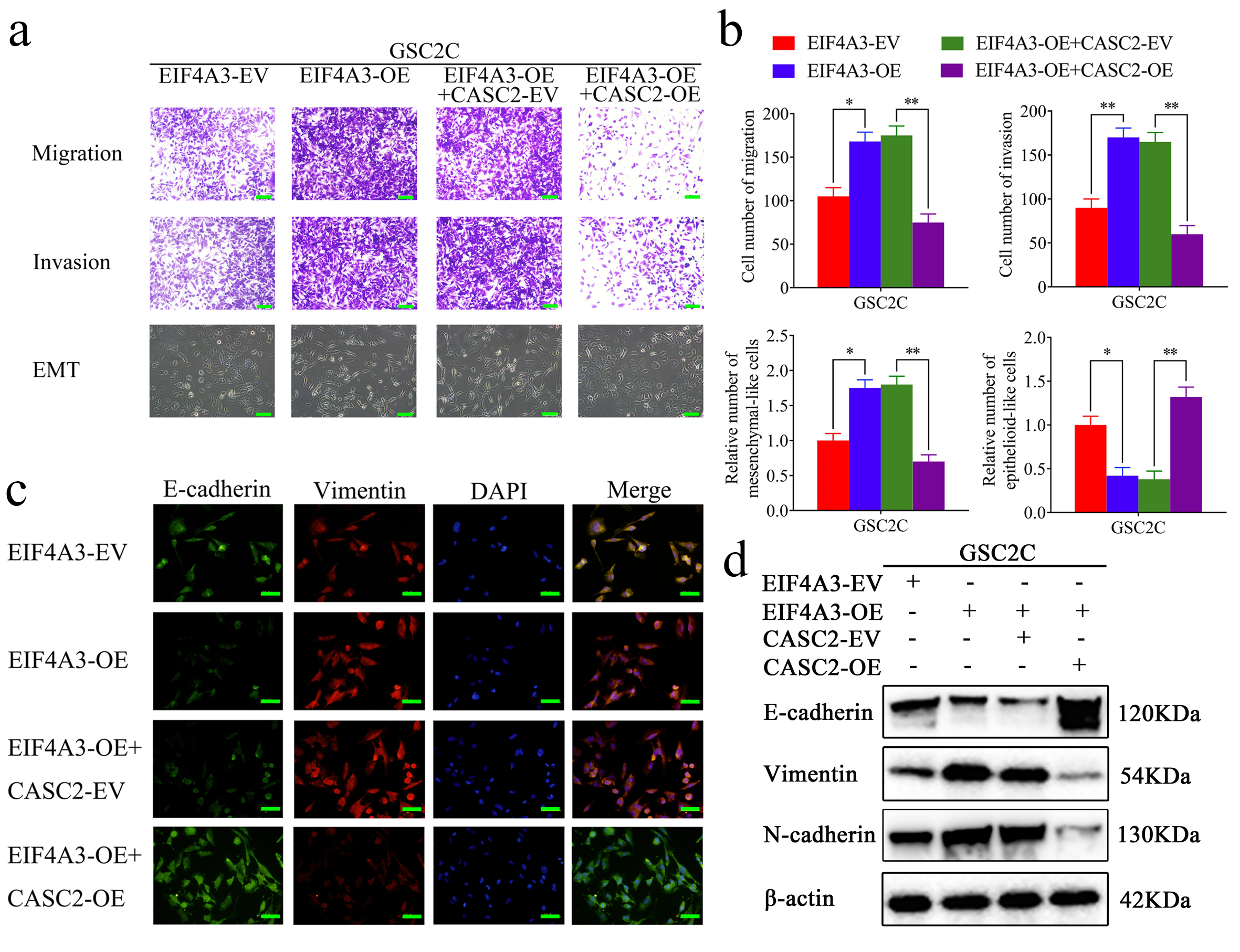

Supplement: Supplementary Figure 7 — EIF4A3 induced the migration, invasion, and EMT of GBM, and these induction effects were restrained following CASC2 overexpression. (A, B) Representative migration assay and Transwell assay showing the migration rates and invasion rates of GSC2C with EIF4A3 overexpression, while reversed after CASC2 overexpression. Scale bar = 100 μm. Representative microphotographs showing the morphological changes in EIF4A3-overexpressed GSC2C, while also reversed after CASC2 overexpression. Scale bar = 50 μm. (C, D) Representative immunofluorescence staining and western blotting showing the changes in E-cadherin, vimentin, and N-cadherin in GSC2C after EIF4A3 overexpression, while reversed after CASC2 overexpression. Scale bar = 50 μm. EV, empty vector; OE, overexpression; NC, negative control; KD, knockdown. All data are expressed as the mean ± SD (three independent experiments). *P < 0.05; **P < 0.01; ***P < 0.001. [file Image_7.tif]

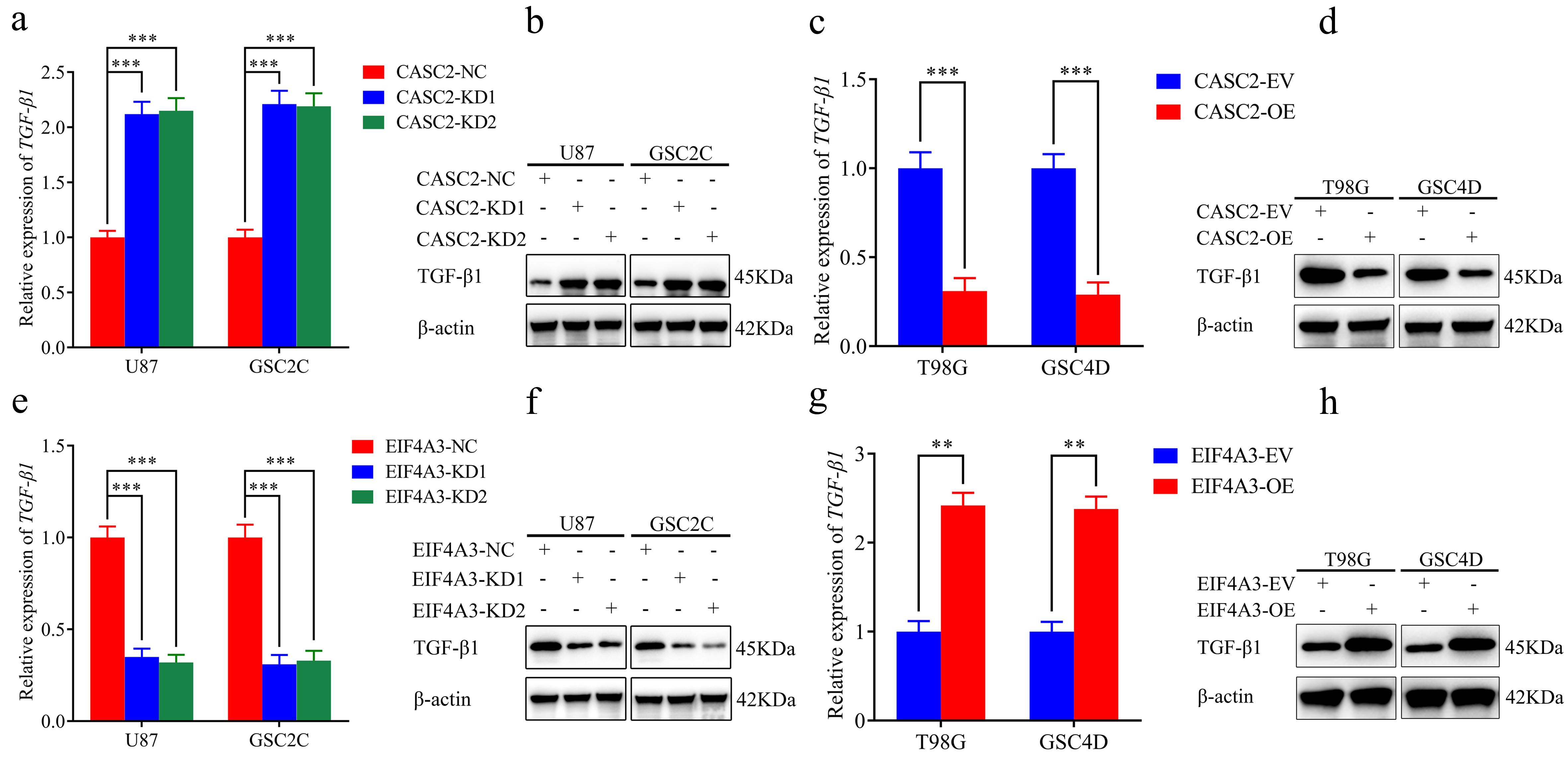

Supplement: Supplementary Figure 8 — The expression of TGF-β1 after CASC2 or EIF4A3 knockdown or overexpression. (A, B) qPCR (A) and western blotting (B) showing the expression of TGF-β1 increased in U87 cells and GSC2C after CASC2 knockdown. (C, D) qPCR (C) and western blotting (D) showing the expression of TGF-β1 decreased in T98G cells and GSC4D after CASC2 overexpression. (E, F) qPCR (E) and western blotting (F) showing the expression of TGF-β1 decreased in U87 cells and GSC2C after EIF4A3 knockdown. (G, H) qPCR (G) and western blotting (H) showing the expression of TGF-β1 increased in T98G cells and GSC4D after EIF4A3 overexpression. EV, empty vector; OE, overexpression; NC, negative control; KD, knockdown. All data are expressed as the mean ± SD (three independent experiments). *P < 0.05; **P < 0.01; ***P < 0.001. [file Image_8.tif]
