# Supplementary material for: The EIF4A3/CASC2/RORA Feedback Loop Regulates the Aggressive Phenotype in Glioblastomas
Source: Front Oncol. 2021 Aug 2;11:699933. doi: 10.3389/fonc.2021.699933 (PMC8366401; doi:10.3389/fonc.2021.699933)
Supplement: Supplementary file 9 [file Table_1.docx]

Supplementary Table 1. Clinical information of the primary glioma stem-like cells

|  | GSC2C | GSC2D | GSC3C | GSC3D | GSC4C | GSC4D |
| --- | --- | --- | --- | --- | --- | --- |
| Gender | Male | Female | Female | Male | Male | Female |
| Age | 65 years old | 53 years old | 60 years old | 46 years old | 64 years old | 58 years old |
| Location | Left frontal lobe | Right insula | Right frontal lobe | Left parietal lobe | Right occipital lobe | Right temporal lobe |
| Pathological diagnosis | Astrocytoma | Astrocytoma | Anaplastic astrocytoma | Anaplastic astrocytoma | Glioblastoma | Glioblastoma |
| WHO grade | II | II | III | III | IV | IV |
| Ki-67 | 35% (+) | 40% (+) | 50% (+) | 55% (+) | 60% (+) | 60% (+) |
