# Supplementary material for: The EIF4A3/CASC2/RORA Feedback Loop Regulates the Aggressive Phenotype in Glioblastomas
Source: Front Oncol. 2021 Aug 2;11:699933. doi: 10.3389/fonc.2021.699933 (PMC8366401; doi:10.3389/fonc.2021.699933)
Supplement: Supplementary file 10 [file Table_2.docx]

**Supplementary Table 2.** Relationship of RORA expression to clinical features of glioma patients

| **Clinical features** | | **Samples**  **(*n* = 87)** | **RORA expression*** | | ***P* value** |
| --- | --- | --- | --- | --- | --- |
|  |  |  | **Low (*n* = 35)** | **High (*n* = 52)** |  |
| **Sex** | Male | 48 | 20 | 28 | P=0.762 |
|  | Female | 39 | 15 | 24 |  |
| **Age** | ≤ 50 | 37 | 14 | 23 | P=0.695 |
|  | > 50 | 50 | 21 | 29 |  |
| **WHO grade** | LGG | 45 | 8 | 37 | **P＜0.001** |
|  | GBM | 42 | 27 | 15 |  |

*: RORA expression was detected by immunohistochemistry and evaluated according to the German immunohistochemical score. High expression was defined as score ≥4.
