# Supplementary material for: The EIF4A3/CASC2/RORA Feedback Loop Regulates the Aggressive Phenotype in Glioblastomas
Source: Front Oncol. 2021 Aug 2;11:699933. doi: 10.3389/fonc.2021.699933 (PMC8366401; doi:10.3389/fonc.2021.699933)
Supplement: Supplementary file 11 [file Table_3.docx]

| **Primer** | **Forward (5’-3’)** | **Reverse (5’-3’)** |
| --- | --- | --- |
| RORA-KD1 | GAUGUGUGGUGCUAGACAAGU | UUGUCUAGCACCACACAUCAG |
| RORA -KD2 | GGAGAAGUCAGCAAAGCAAUG | UUGCUUUGCUGACUUCUCCUG |
| CASC2-KD1 | AGACUAUAAUGAUACCUUGGG | CAAGGUAUCAUUAUAGUCUUU |
| CASC2-KD2 | UAAAGACUAUAAUGAUACCUU | GGUAUCAUUAUAGUCUUUAGA |
| EIF4A3-KD1 | AACCUUUAUUCAACAUUUCAU | GAAAUGUUGAAUAAAGGUUUC |
| EIF4A3-KD2 | UUUGAAACCUUUAUUCAACAU | GUUGAAUAAAGGUUUCAAAGA |
| siRNA-NC | UUCUUCGAAGGUGUCACGUTT | ACGUGACACCUUCGAAGAATT |

**Supplementary Table 3. siRNA sequences**
