# Supplementary material for: The EIF4A3/CASC2/RORA Feedback Loop Regulates the Aggressive Phenotype in Glioblastomas
Source: Front Oncol. 2021 Aug 2;11:699933. doi: 10.3389/fonc.2021.699933 (PMC8366401; doi:10.3389/fonc.2021.699933)
Supplement: Supplementary file 12 [file Table_4.docx]

**Supplementary Table 4. RT-qPCR Primers**

| **Primer** | **Forward (5’-3’)** | **Reverse (5’-3’)** |
| --- | --- | --- |
| RORA | ACTCCTGTCCTCGTCAGAAG | CATCCCTACGGCAAGGCATTT |
| TGFβ1 | GGCCAGATCCTGTCCAAGC | GTGGGTTTCCACCATTAGCAC |
| E1F4A3 | GGGGCATCTACGCTTACGG | GCGATGACATCTCTCCCTTTGA |
| β-actin | CATGTACGTTGCTATCCAGGC | CTCCTTAATGTCACGCACGAT |
| CASC2 | CCCAGTGTCTTGCCCTTAGG | GCTTGTGCCTCTGTTTGCTC |
| U6 | TGACCCTTAAGTACCCCATCGA | TTGTAGAAGGTGTGGTGCCAGAT |
